# The Long Noncoding RNA *ΒFaar* Promotes White Adipose Tissue Browning and Prevents Diet‐Induced Obesity

**DOI:** 10.1002/advs.202505545

**Published:** 2025-06-23

**Authors:** Yue Yang, Bin Huang, Baixue Sha, Danni Gao, Yimeng Qin, Ziyi Li, Xi Chen, Yinuo Jin, Yi Pan, Yanfeng Zhang, Yumeng Shen, Yu Liu, Liang Jin, Fangfang Zhang

**Affiliations:** ^1^ State Key Laboratory of Natural Medicines, Jiangsu Key Laboratory of Druggability of Biopharmaceuticals, School of Life Science and Technology China Pharmaceutical University 24 Tongjiaxiang Nanjing China; ^2^ Jiangsu Key Laboratory of TCM Evaluation and Translational Research, School of Traditional Chinese Pharmacy China Pharmaceutical University Nanjing 211198 China; ^3^ Endocrinology Department Sir Run Run Hospital of Nanjing Medical University 109# Longmian Road Nanjing 210000 China; ^4^ Nanjing HanKai Academy Jiangpu Street, Pukou Nanjing Jiangsu Province China

**Keywords:** browning, lipid droplets, lncRNA, obesity, white adipose tissue

## Abstract

The conversion of white adipose tissue (WAT) to brown adipose tissue (BAT) is a promising strategy for obesity treatment. It is previously identified *βFaar* as a conserved long noncoding RNA (lncRNA) regulator of islet β‐cell function in individuals with obesity, but its effect on WAT browning is not well understood. In this study, it is discovered that *βFaar* expression in adipose tissue markedly decreases with the progression of obesity in both mice and humans. *βFaar* in adipose tissue reduces lipid droplet (LD) size in WAT and promotes a browning phenotype in inguinal WAT (iWAT), leading to the amelioration of high‐fat diet (HFD)‐induced obesity. These effects can be attributed to crosstalk between *βFaar* and proteins within the master regulatory pathways of LD formation and WAT browning, including RAS oncogene family 18 (RAB18) and interferon regulatory factor 4 (IRF4). Specifically, *βFaar* inhibits LD swelling by binding to RAB18 and promoting IRF4 nuclear translocation, increases uncoupling protein 1 (UCP1) transcription, and further induces iWAT browning by binding to karyopherin subunit alpha 6 (KPNA6). Together, these results demonstrate the critical roles of *βFaar* in regulating iWAT browning and preserving metabolic health; thus, *βFaar* may be a potential therapeutic target for management of obesity and related disorders.

## Graphical abstract

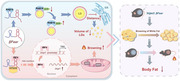

## 1 Introduction

Adipose tissue has traditionally been categorized as white adipose tissue (WAT) or brown adipose tissue (BAT) depending on its morphology and function.^[^ ^1^ ^]^ WAT contains large, unilocular lipid droplets (LDs) and is involved in lipid storage. However, excessive lipid storage in WAT results in obesity and related metabolic disorders, including insulin resistance and type 2 diabetes (T2D).^[^ ^2^ ^]^ In contrast, BAT contains small, multilocular LDs, specializes in energy expenditure and thermogenesis, and is an attractive target for obesity treatment.^[^ ^3^ ^]^ BAT is densely packed with mitochondria expressing high levels of uncoupling protein 1 (UCP1), which facilitates proton leakage to uncouple respiration from ATP synthesis.^[^ ^4^ ^]^ In rodents, BAT is activated by overfeeding as a physiological response to limit weight gain.^[^ ^5^ ^]^ In humans, BAT was initially thought to be present only in infants, but substantial depots of UCP1‐expressing brown‐like fat has recently been detected in adults,^[^ ^6^ ^]^ and the activity of this tissue is positively correlated with the resting metabolic rate and negatively correlated with body mass index (BMI).^[^ ^7^ ^]^

Recent research has identified a third type of adipose tissue, termed inducible brown‐like fat or beige adipose tissue, which can be induced from WAT due to exercise, diet, and exposure to cold or various activators.^[^ ^8^ ^]^ Under basal conditions, brown‐like fat expresses little to no UCP1, but the induction of UCP1 expression in response to cold promotes thermogenesis and energy expenditure.^[^ ^9^ ^]^ Interestingly, brown‐like fat in adult humans has a molecular signature that resembles that of rodent beige fat, and increasing WAT browning in rodents increases energy expenditure and suppresses diet‐induced obesity and glucose intolerance.^[^ ^10^ ^]^ On the other hand, WAT browning can be inhibited by deleting *Prdm16*, a transcriptional cofactor that increases UCP1 expression and promotes obesity and severe insulin resistance.^[^ ^11^ ^]^ Thus, a deeper understanding of the mechanisms governing WAT browning is very important, as it may lead to the development of strategies to improve metabolic health.

Long noncoding RNAs (lncRNAs) are a subset of transcripts that are longer than 200 nt in length but lack the potential to encode proteins. Compared with protein‐coding genes, lncRNAs display greater cell‐type specificity.^[^ ^12^, ^13^ ^]^ Thus, identifying the modes of action of dysregulated, cell‐specific lncRNAs may provide insights into how metabolic disturbances occur in a cell‐dependent manner and guide the development of cell‐specific therapeutics. Several lncRNAs have recently emerged as important regulators of WAT browning. One of them, *LINC00473*, was shown to interact with mitochondrial and LD proteins, thereby altering cellular metabolism.^[^ ^14^ ^]^ The lncRNA Lexis is regulated by PPARγ, and Lexis knockdown promotes adipose tissue begging.^[^ ^15^ ^]^ Although some lncRNAs have been shown to be central regulators of the brown adipogenesis program, the lncRNA regulatory network is incompletely understood, and further studies are needed to fully understand the regulatory roles of lncRNAs in WAT browning and develop therapeutic approaches for obesity and obesity‐related diseases.

In a previous study, we identified the long noncoding RNA *βFaar*, which can regulate β‐cell function and apoptosis in obese mice.^[^ ^16^ ^]^ Here, we found that *βFaar* is markedly downregulated in the WAT of obese mice. Next, the role of *βFaar* in adipose tissue was assessed in male mice with WAT‐specific *βFaar* knockout and introduction of the pHAGE‐CMV vector, which leads to *βFaar* overexpression mediated by the *Adipoq* promoter. We demonstrated that *βFaar* inhibits LD enlargement in WAT and promotes inguinal WAT (iWAT) browning by increasing energy expenditure to alleviate obesity. Mechanistically, *βFaar* attenuates LD formation and promotes adipocyte browning through its protein partners RAS oncogene family 18 (RAB18) and interferon regulatory factor 4 (IRF4) in a cell‐specific manner. Our results highlight the important role of *βFaar* in obesity prevention and provide potential therapeutic targets for the treatment of obesity‐associated metabolic diseases.

## 2 Results

### 2.1 βFaar Expression Markedly Decreases as Obesity Progresses

**Figure 1:**
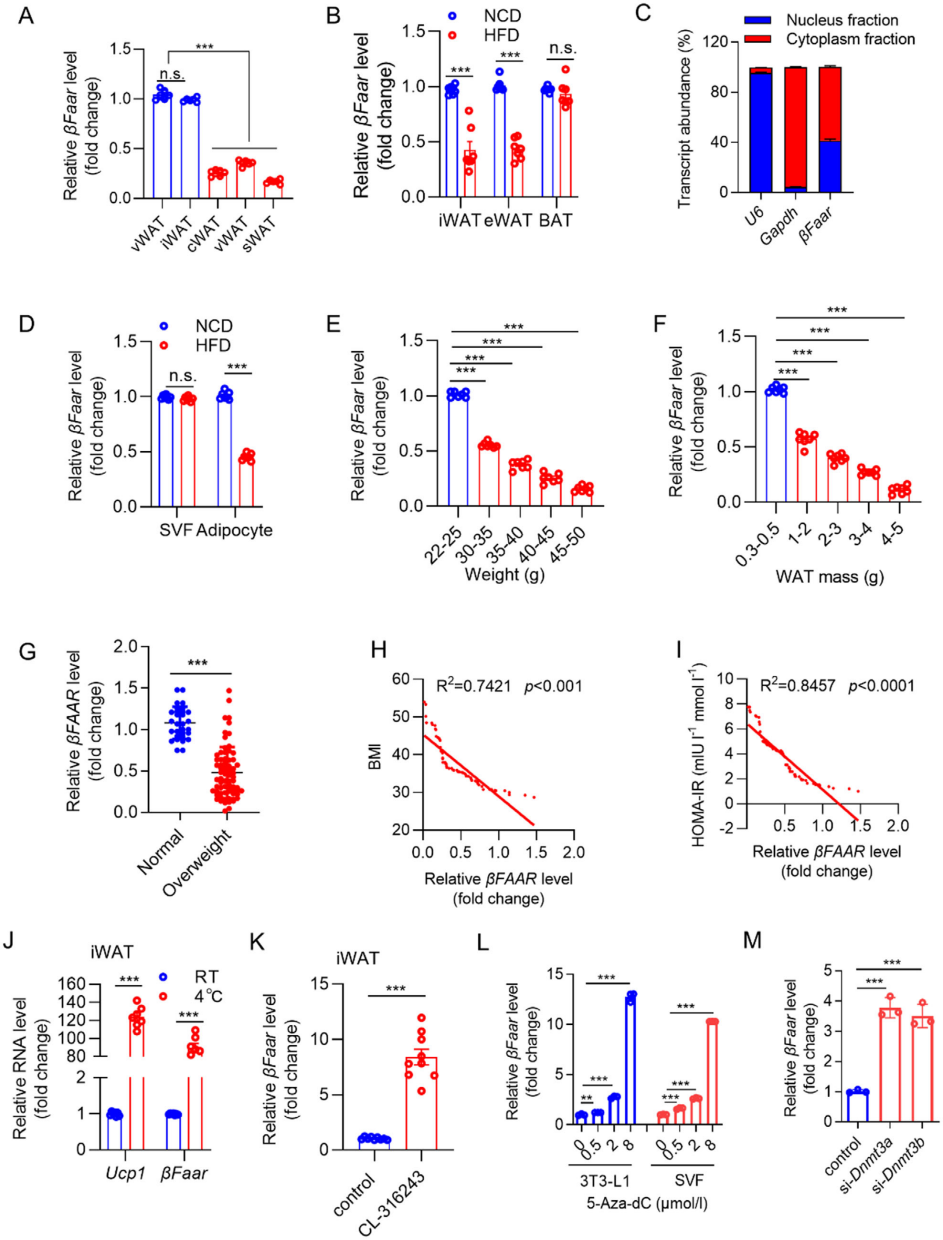
*βFaar* expression markedly decreases as obesity progresses. A) The expression of *βFaar* in different locations of adipose tissue was quantified via qRT‐PCR (*n* = 6 mice). B) The expression of *βFaar* in the iWAT, eWAT, and BAT of mice fed a normal chow diet (NCD) or high‐fat diet (HFD) was quantified via qRT‒PCR (*n* = 7 mice). C) The expression of *βFaar* in the WAT nuclear and cytoplasmic fractions. *βFaar*, *Gapdh*, and *U6* small nuclear RNA levels in the purified nuclear and cytoplasmic fractions of WAT were detected via qRT‐PCR. D) The expression levels of *βFaar* in SVF and primary adipocytes isolated from mice were quantified via qRT‐PCR (*n* = 7 mice). (E, F) Expression levels of *βFaar* at different stages during the development of obesity in HFD‐fed mice based on weight E) and WAT mass F) (*n* = 7 mice). G–I) Expression levels of *βFAAR* in human subcutaneous adipose tissue samples were categorized as normal (*n* = 30 individuals) or obese and IR (*n* = 70 individuals). Scatter plots depicting correlations among *βFAAR* expression and BMI H) and HOMA‐IR I). J) Analysis of the expression patterns of *βFaar* and *Ucp1* in iWAT after 24 h of cold exposure (*n* = 7 mice). K) Expression of *βFaar* in iWAT treated with the β3 adrenoceptor agonist CL‐316243 (*n* = 9 mice). L) 3T3‐L1 and SVF cells were treated with 5‐Aza‐dC, qRT‐PCR was used to test the *βFaar* levels. M) *si‐Dnmt3a* or *si‐Dnmt3b* was transfected into SVF cells isolated from obese mice, qRT‐PCR was used to test the *βFaar* levels (*n* = 3 mice). The fold change values were calculated via the 2^−ΔΔCt^ method. The data are presented as the means ± SEMs. The *p* values obtained using a two‐tailed unpaired Student's *t*‐test or two‐way ANOVA are indicated; ***p < 0.001.

*Mus‐βFaar* has only one variant while *Has‐βFAAR* has four variants, among which *Has‐βFAAR* transcript variant 4 (*βFAAR‐V4*) is highly expressed in human adipose tissue (Figure S1A, Supporting Information). Consistent with the findings of our previous study, *βfaar* expression in WAT was greater than that in other tissues except the pancreas and kidney, and was not different between male and female mice (Figure S1B, Supporting Information). To explore the role of *βFaar* in adipose tissue, we examined its expression profile in various WATs. Quantitative real‐time PCR (qRT‐PCR) revealed that *βFaar* was more abundant in visceral WAT (vWAT) and iWAT than in other WAT types (**Figure** **1**A). We next examined whether *βFaar* expression in adipose tissue is altered in obese mice. *βFaar* expression was substantially decreased in the WATs of high‐fat diet (HFD)‐fed and genetically obese (*ob/ob*) mice (Figure 1B; Figure S1C, Supporting Information), whereas *βFaar* expression was not different between the gonad WAT (gWAT) and iWAT of male and female mice fed an HFD (Figure S1D, Supporting Information). Subcellular fractionation analysis revealed that *βFaar* expression was higher in the cytoplasm than in the nucleus in WAT (Figure 1C). Notably, this obesity‐associated reduction in *βFaar* expression was restricted to adipocytes and was not observed in the stromal vascular fraction (SVF) (Figure 1D). In addition, *βFaar* expression was strongly associated with weight gain and WAT mass in HFD‐fed (Figure 1E,F) and *ob/ob* mice (Figure S1E,F, Supporting Information). Notably, *βFAAR‐V4* expression tends to decrease in adipose tissue in obese humans (Figure 1G), and *βFAAR‐V4* expression in the WAT of obese individuals is negatively correlated with BMI and homeostasis model assessment of insulin resistance (HOMA‐IR) (Figure 1H,I).

Owing to the essential role of iWAT in adaptive thermogenesis, the expression level of *βFaar* under thermogenic stimulation was explored. *βFaar* was upregulated in iWAT upon cold exposure (4 °C), showing an expression pattern similar to that of the thermogenic gene UCP1 (Figure 1J). To further confirm the involvement of *βFaar* in thermogenesis, the β3 adrenoceptor agonist CL‐316243 was applied. As expected, *βFaar* was also upregulated upon activation of the β3 adrenergic signaling pathway in vivo (Figure 1K). Surprisingly, the expression of *βFaar* in eWAT increased slightly after cold exposure and CL‐316243 stimulation (Figure S1G,H, Supporting Information) even though eWAT is rarely involved in adaptive thermogenesis, suggesting that *βFaar* could participate in thermogenesis in WAT. Moreover, treating 3T3‐L1 and SVF cells with 5‐Aza‐dC increased the expression of *βFaar* in a dose‐dependent manner (Figure 1L). Furthermore, rescue experiments demonstrated that the palmitate‐induced downregulation of *βFaar* was reversed when SVF cells isolated from HFD‐fed mice were transfected with *siDnmt3a* and *siDnmt3b* (Figure 1M). Collectively, these observations suggest that decreased *βFaar* expression induced by DNA methylation may contribute to adipose tissue dysfunction in individuals with obesity, alternatively, that *βFaar* may facilitate adipose adaptation to nutritional stress to preserve metabolic health.

### 2.2 βFaar Alleviates HFD‐Induced Obesity and Ameliorates Metabolic Dysfunction

**Figure 2:**
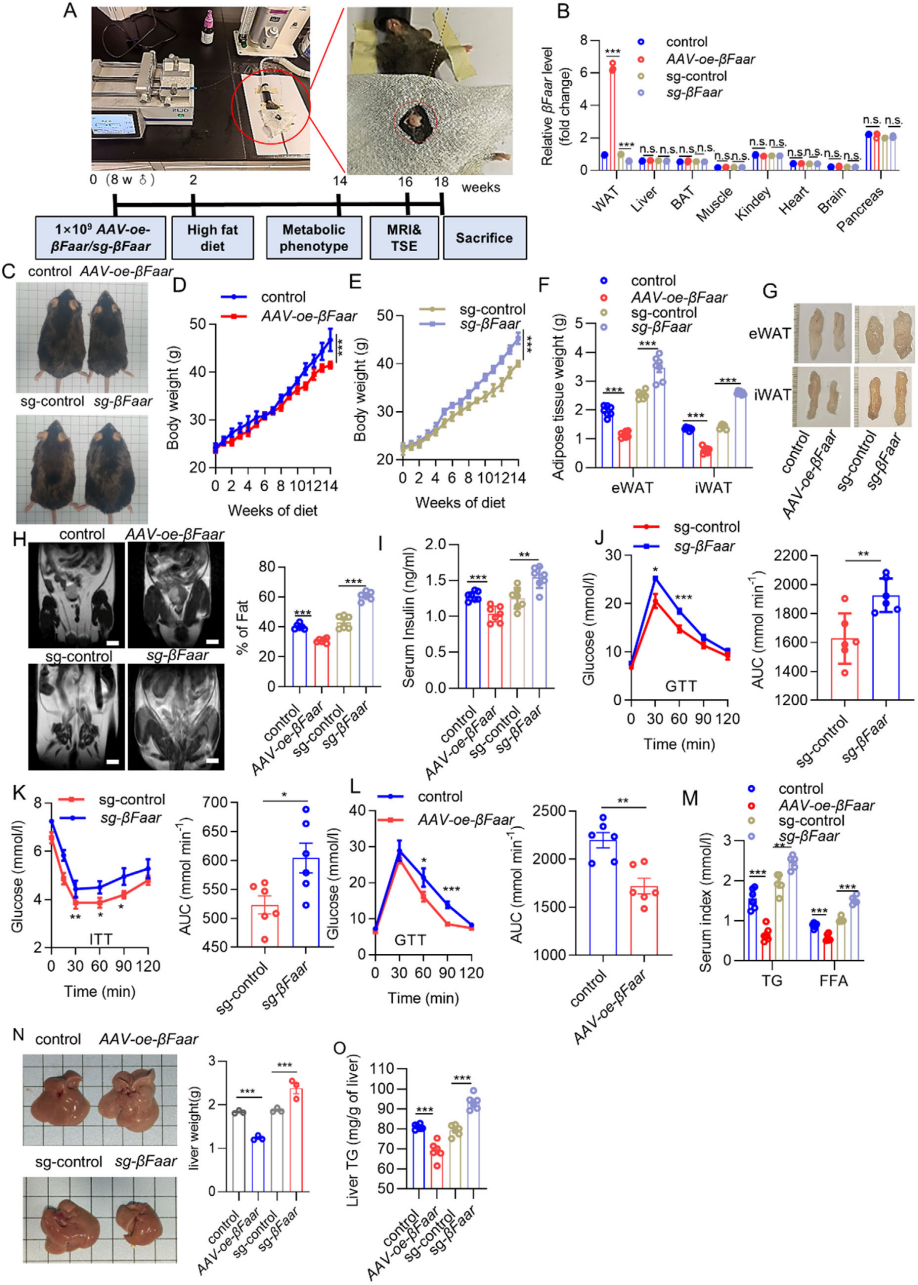
*βFaar* alleviates HFD‐induced obesity and ameliorates metabolic dysfunction. A) The flowchart outlining the in vivo experiments designed to assess adipocyte function through fat pad in situ injection of *AAV‐oe‐βFaar* or *sg‐βFaar* (*n* = 10 mice). Eight‐week‐old male *AAV‐oe‐βFaar*, *sg‐βFaar* mice, and control mice (AAV‐*Adipoq* promoter‐pHAGE/pCas‐Puro‐U6) were subjected to 18‐week high‐fat diet (HFD). B) The expression level of *βFaar* in various tissues was quantified using qRT‐PCR (*n* = 3 mice). C) Representative photos of control, *AAV‐oe‐βFaar*, and *sg‐βFaar* fed with either HFD for 18 weeks (*n* = 3 mice). D,E) Dynamic changes in body weight of *AAV‐oe‐βFaar* (D, *n* = 9 mice) *sg‐βFaar* (E, *n* = 6 mice) mice during 14 weeks of HFD diet. H) Representative coronal section MRI images and visceral and subcutaneous adipose tissue volume of HFD‐fed control, *AAV‐oe‐βFaar*, and *sg‐βFaar* mice (*n =* 3 mice). I) Serum insulin content was measured (*n* = 7 mice). J,K) intraperitoneal glucose tolerance test (IPGTT; 1.5 g kg^−1^) (J, *n* = 6 mice), and intraperitoneal insulin tolerance test (IPITT; 0.75 U kg^−1^, K, *n* = 6 mice) were performed on both *sg‐βFaar* mice and control mice at week 14 of HFD administration. The corresponding area under the curve (AUC) for blood glucose levels was calculated. (L) IPGTT (1.5 g kg^−1^) was performed on both *AAV‐oe‐βFaar* mice and control mice at week 14 of HFD administration (*n* = 6 mice). The corresponding area under the curve (AUC) for blood glucose levels was calculated. M) Serum triglyceride (TG) and non‐esterified fatty acid (NEFA) levels were analyzed after overexpression of *βFaar* in mice by using biochemical testing reagents (*n* = 7 mice). N) Liver weight was calculated of *AAV‐oe‐βFaar* mice, *sg‐βFaar* mice, and control mice (*n* = 3 mice), scale 1 cm × 1 cm. O) Hepatic TG content was determined in HFD‐treated mice expressing *AAV‐oe‐βFaar* (*n* = 7 mice). mRNA fold change values were calculated via the 2^−ΔΔCt^ method. The data are presented as the means ± SEMs. The *p* values obtained from two‐tailed unpaired Student's *t*‐test or two‐way ANOVA are indicated; **p < 0.01, ***p < 0.001.

We subsequently constructed adeno‐associated virus (AAV) particles to induce adipocyte‐specific *βFaar* overexpression or knockdown using the *adiponectin* promoter to determine the role of *βFaar* in adipose tissue under physiological conditions. First, 1 × 10^9^ recombinant control, *AAV‐oe‐βFaar* particles or *sg‐βFaar* particles were used to infect the eWAT and iWAT of mice via in situ injection, as depicted in the schematics of the surgical procedure in **Figures** **2**A and S2A (Supporting Information). Hereafter, we refer to these model mice as adipose‐specific *βFaar*‐overexpressing (*AAV‐oe‐βFaar*) and adipose‐specific *βFaar*‐knockdown (*sg‐βFaar*) mice. *βFaar* was upregulated 6‐fold and downregulated by 50% in the WAT of *AAV‐oe‐βFaar* and *sg‐βFaar* mice, respectively, compared with that in the WAT of control mice, but there was no difference in *βFaar* expression in the other organs (Figure 2B). The body weights of the *AAV‐oe‐βFaar* and control mice fed a normal chow diet (NCD) did not differ significantly (Figure S2B, Supporting Information). Conversely, *βFaar* overexpression attenuated HFD‐induced body weight gain, whereas knockdown of *βFaar* in WAT exacerbated diet‐induced body weight gain (Figure 2C–E) when food intake was comparable (Figure S2C, Supporting Information). The total body fat mass of the *sg‐βFaar* mice was significantly greater than that of the control mice, but no change in lean mass was observed, whereas *AAV‐oe‐βFaar* mice had a significantly lower total body fat mass than that of the control mice (Figure S2D, Supporting Information). Mice with adipocyte‐specific *βFaar* knockdown exhibited significant enlargement of the eWAT and iWAT (Figure 2F,G). Magnetic resonance imaging (MRI) confirmed that whole‐body adiposity was significantly decreased in the *AAV‐oe‐βFaar* mice fed an HFD but increased in the *sg‐βFaar* mice compared to each control (Figure 2H). We also evaluated obesity‐related metabolic parameters after 14 weeks of HFD feeding and revealed that the blood glucose and fasting serum insulin levels were significantly lower in *AAV‐oe‐βFaar* mice than in control mice (Figure 2I; Figure S2E,F, Supporting Information).

Similarly, the glucose tolerance test (GTT) and insulin tolerance test (ITT) indicated that HFD‐fed *sg‐βFaar* mice developed more severe glucose intolerance and insulin resistance (Figure 2J,K), whereas *AAV‐oe‐βFaar* mice exhibited improved glucose intolerance and insulin resistance (Figure 2L; Figure S2G, Supporting Information). In addition, the serum levels of triglycerides (TGs) and free fatty acids (FFAs) decreased when *βFaar* was overexpressed, and the opposite phenomena were observed in *sg‐βFaar* mice (Figure 2M). Moreover, the accumulation of lipids in the liver, which commonly occurs with obesity‐related metabolic disorders, was also investigated. We found that the liver weight and liver fat content were significantly greater and that hepatic steatosis was more severe in HFD‐fed *sg‐βFaar* mice compared with control mice and that these changes were alleviated in *AAV‐oe‐βFaar* mice fed an HFD (Figure 2N,O; Figure S2H, Supporting Information). We also investigated changes in skeletal muscle‐related functions, but there were no significant changes in terms of muscle morphology, plasma creatine kinase (CK) content, or muscle TG content (Figures S2I‐S2K, Supporting Information). These results implied that *βFaar* knockdown in adipose tissue could exacerbate obesity‐induced metabolic dysfunction.

### 2.3 βFaar Enhances Energy Expenditure and Adaptive Thermogenesis

**Figure 3:**
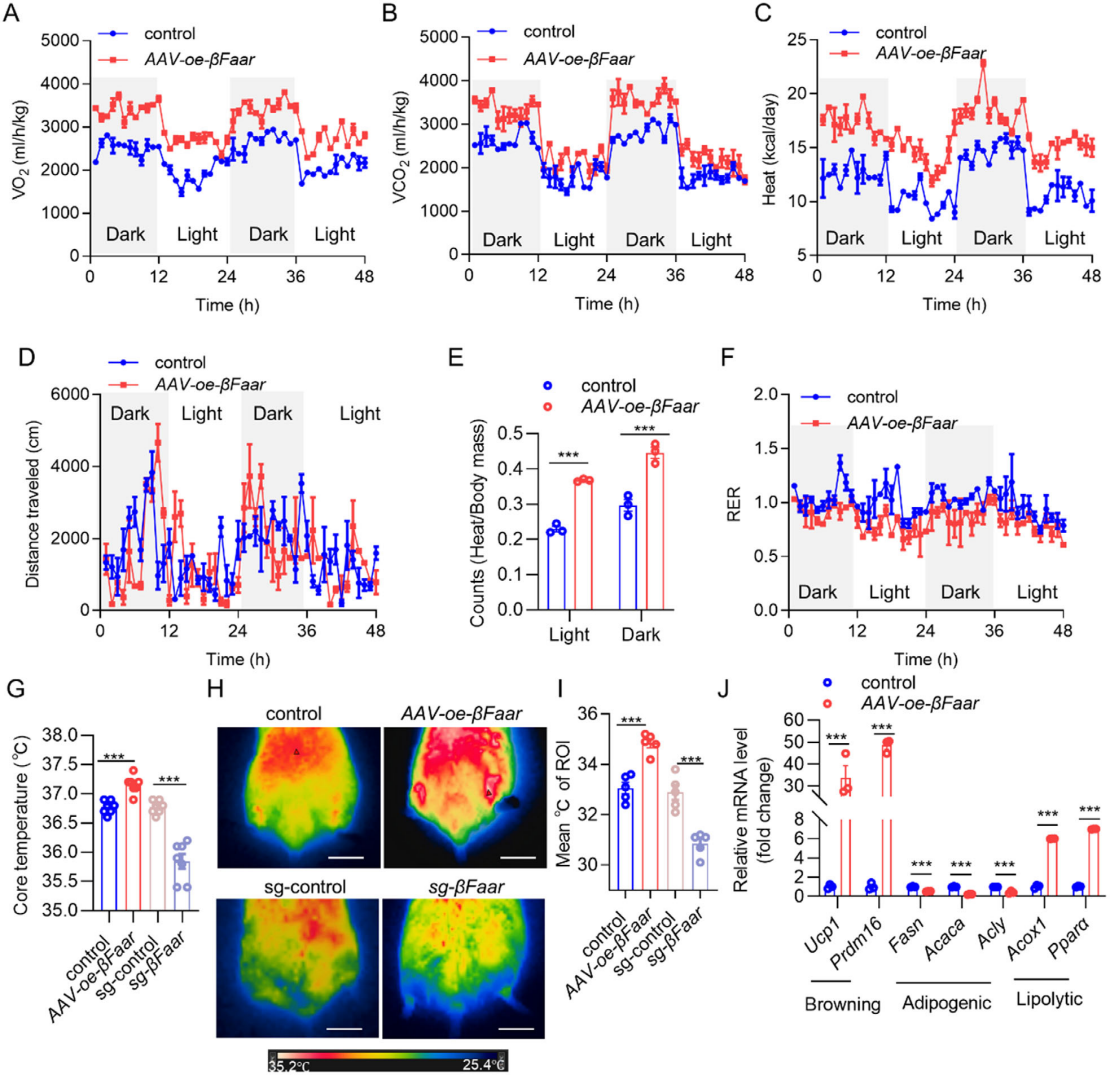
*βFaar* enhances energy expenditure and adaptive thermogenesis. A–D) The variables of interest, including VO_2_ A), VCO_2_ B), heat production C), and average values of physical activity D) were conducted by *AAV‐oe‐βFaar*. The day/night bar represents a 12 h duration. VO_2_, VCO_2,_ and heat production were analyzed by ANCOVA with total body mass as a covariate (*n* = 3 mice). E) Heat production was calculated using different body weights as controls (*n* = 3 mice). F) The ratio of VCO_2_/VO_2_ was analyzed by *AAV‐oe‐βFaar*. The day/night bar represents a 12 h duration (*n* = 3 mice). G) Rectal temperature of mice was monitored of *AAV*‐*oe*‐*βFaar* mice and *sg*‐*βFaar* mice (*n* = 7 mice). H) Changes in mice body temperature were detected in *AAV‐oe‐βFaar* mice and *sg*‐*βFaar* mice through infrared thermal imaging (IIR) (*n* = 5 mice). The mean °C of ROI (I, *n* = 5 mice). J) Relative mRNA levels of thermogenic, adipogenic, and lipolytic genes were measured in *AAV‐oe‐βFaar* mice and the control group was quantified using qRT‐PCR (*n* = 3 mice). The fold change in mRNA expression was calculated via the 2^−ΔΔCt^ method. The data are presented as the means ± SEMs. The *p* values obtained using a two‐tailed unpaired Student's *t*‐test or two‐way ANOVA are indicated; ***p < 0.001.

As the weight differences between *AAV‐oe‐βFaar*, *sg‐βFaar*, and control mice were not attributable to differences in food intake, we hypothesized that *βFaar* might alleviate adiposity by increasing energy expenditure. Upon assessing energy consumption, we found that, compared with control mice, *AAV‐oe‐βFaar* mice fed an HFD presented increases in oxygen consumption (VO_2_), carbon dioxide production (VCO_2_), and heat generation but not ambulatory movement (**Figure** **3**A–D). These data were also analyzed using total body mass as the covariate, and similar results were obtained (Figure 3E). The respiratory exchange ratio (RER) of the *AAV‐oe‐βFaar* mice was lower than that of the controls, indicating their use of fat as an energy source (Figure 3F). After *βFaar* was knocked down using single‐guide RNA (*sgRNA*), the mice exhibited the opposite phenotype in terms of energy expenditure when fed an HFD. *sg‐βFaar* mice presented a lower VO_2_ and VCO_2_ and less heat generation during both the light and dark cycles (Figure S3A–C, Supporting Information), although the RER (VCO_2_/VO_2_) of these mice was similar to that of the control mice (Figure S3D, Supporting Information). Furthermore, no changes in locomotor activity were observed (Figure S3E, Supporting Information). Notably, after normalization of the VO_2_, VCO_2,_ and heat generation on the basis of lean mass, these parameters were still significantly lower in *sg‐βFaar* mice fed an HFD than in control mice (Figure S3F, Supporting Information).

Consistently, we found that at an ambient temperature of 22 °C, the core temperature of the *AAV‐oe‐βFaar* mice was significantly higher whereas the core temperature of *sg‐βFaar* mice was lower than that of the vehicle‐treated control mice (Figure 3G). In addition, the temperature in the interscapular area, particularly the inguinal area, was significantly greater in *AAV‐oe‐βFaar* mice than in control mice (Figure 3H,I). Further analysis revealed that the mRNA expression of thermogenic genes (*Ucp1* and *Prdm16*) in iWAT was induced by *βFaar* overexpression in HFD‐fed mice, whereas the expression of adipogenic genes (*Fasn, Acaca* and *Acly*) and lipolytic genes (*Pparα* and *Acox1*) varied (Figure 3J), and the opposite phenomena were observed in the WAT of *sg‐βFaar* mice (Figure S3G,H, Supporting Information). These data indicated that *βFaar* overexpression in eWAT and iWAT promoted energy expenditure and adaptive thermogenesis, whereas *βFaar* deficiency may reduce energy consumption and decrease catabolic rates.

### 2.4 βFaar Reduces LD Size and Promotes WAT Browning

**Figure 4:**
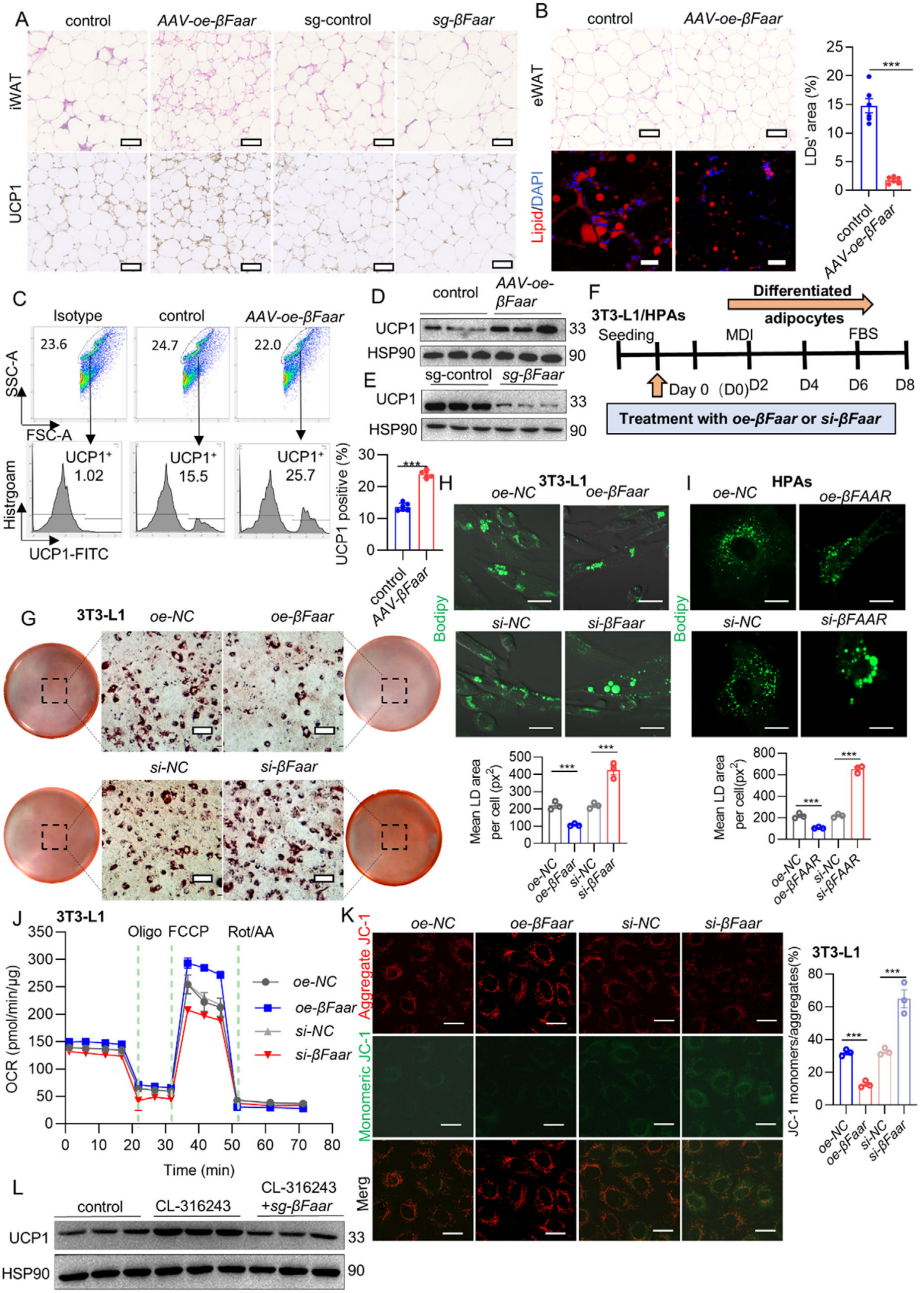
*βFaar* reduces LD size and promotes WAT browning. A) The images illustrating H&E staining and UCP1 immunohistochemistry in iWAT sections treated with *AAV‐oe‐βFaar*, *sg‐βFaar* or control were captured with a scale bar of 100 µm (*n* = 7 mice). B) The images illustrating H&E staining and lipid immunofluorescence in eWAT sections treated with *AAV‐oe‐βFaar* or control were obtained, the LDs’ area (%) was calculated in the right. H&E staining with a scale bar of 100 µm, lipid immunofluorescence with a scale bar of 20 µm (*n* = 7 mice). C) Flow cytometry assay was employed to detect the content of UCP1^+^ cells in iWAT after treatment with *AAV‐oe‐βFaar* or control (*n* = 7 mice). D,E) Immunoblotting analysis revealed representative images displaying the expression levels of UCP1 in iWAT of *AAV‐oe‐βFaar* mice (D, *n* = 6 mice) or *sg‐βFaar* mice (E, *n* = 6 mice). F) The flowchart outlining the experimental design for assessing *βFaar* function in 3T3‐L1 cells and HPA cells (*n* = 7 mice). G) Oil red O staining was performed to evaluate the number of LDs in 3T3‐L1 cells transfected with *oe/si‐βFaar* or control, with a scale bar set at 20 µm. H,I) Representative images showing LDs labeled by green fluorescence were observed in *oe/si‐βFaar* treated 3T3‐L1 differentiated cells (H, 1 × 10^4^ cells per confocal dish) and in the *oe/si‐βFAAR* treated HPAs differentiated cells (I, 1 × 10^4^ cells per confocal dish), with a scale bar set at 20 and 10 µm respectively. The quantification of bodipy is listed in below. J) Oxygen consumption rate (OCR) measurement was conducted on 3T3‐L1 cells following *oe/si‐βFaar* transfection. K) 3T3‐L1 cells (left panel) and statistical analysis results (right panel) after transfection with *oe/si‐βFaar* and staining of mitochondria with JC‐1 to assess MMP with a scale bar of 20 µm (1 × 10^5^ cells per confocal dish). L) Immunoblotting analysis revealed representative images displaying the expression levels of UCP1 upon treatment with CL‐316243 and *sg‐βFaar*. The fold change in mRNA expression was calculated via the 2^−ΔΔCt^ method. The data are presented as the means ± SEMs. The *p* values obtained using a two‐tailed unpaired Student's *t*‐test or two‐way ANOVA are indicated; ***p < 0.001.

As the decreased adiposity and increased energy expenditure in HFD‐fed *AAV‐oe‐βFaar* mice were not attributable to changes in food intake or ambulatory activity, we examined whether *AAV‐oe‐βFaar* mice presented increased WAT browning. iWAT from *AAV‐oe‐βFaar* mice exhibited a browning phenotype, with multilocular LDs and increased UCP1 protein expression. In contrast, the abundance of beige adipocytes in iWAT was greatly diminished by *βFaar* knockdown (**Figure** **4**A). We also observed a significant decrease in the size of the LDs (Figure 4B) and hematoxylin and eosin (H&E) staining revealed the accumulation of more fat with LDs that exhibited a unilocular phenotype (Figure S4A, Supporting Information) in the eWAT of *sg‐βFaar* mice. Notably, these phenotypes were not observed in BAT (Figure S4B, Supporting Information). Consistent with these histological changes, the abundance of UCP1^+^ beige adipocytes significantly increased (Figure 4C) and UCP1 was upregulated in the iWAT of *AAV‐oe‐βFaar* mice compared with that in the iWAT of control mice (Figure 4D). Additionally, the number of UCP1^+^ beige adipocytes and UCP1 expression were markedly lower in the iWAT of *sg‐βFaar* mice than in that of control mice (Figure S4C,D, Supporting Information, and Figure 4E). At the transcription level, the expression of several hallmark genes that regulate browning, including peroxisome proliferator activated receptor γ coactivator 1‐α (*Pgc1α*), its known downstream gene UCP1, and the cell death activator CIDE‐A (*Cidea*), was also increased in the WAT of *AAV‐oe‐βFaar* mice and downregulated in the WAT of *sg‐βFaar* mice (Figure S4E, Supporting Information). These results suggest that *βFaar* knockdown suppressed iWAT browning.

The role of *βFaar* in white‐to‐beige adipocyte conversion was also investigated in vitro with primary adipocytes differentiated from 3T3‐L1 preadipocytes and human precursor adipocytes (HPAs). The transfection and differentiation processes used in this study are shown in Figure 4F. The transfection efficiency was confirmed via qRT‒PCR (Figure S4F,G, Supporting Information). Transfection of *oe‐βFaar*, but not *si‐βFaar*, largely blocked adipocyte differentiation, as shown by oil red O staining (Figure 4G; Figure S4H, Supporting Information). Morphological analysis of the intracellular lipids revealed that, compared with control cells, *oe‐βFaar* transfected in 3T3‐L1 cells and HPAs exhibited smaller LDs (Figure 4H–I). Overexpression of *βFaar* also reduced the accumulation of TGs and nonesterified fatty acids (NEFAs) in both 3T3‐L1 cells (Figure S4I,J, Supporting Information) and HPAs (Figure S4K,L, Supporting Information).

Moreover, *βFaar* overexpression in 3T3‐L1 cells inhibited mitochondrial apoptosis and improved mitochondrial function (Figure 4J,K), and the opposite effects were observed when *βFaar* was silenced. Moreover, CL‐316243‐induced UCP1 expression was altered in the iWAT of *sg‐βFaar* mice (Figure 4L). Taken together, these data demonstrate that *βFaar* promoted the white‐to‐beige adipocyte conversion.

### 2.5 βFaar Reduces LD‐Endoplasmic Reticulum (ER) Apposition by Binding to RAB18, Resulting in LD Shrinkage

**Figure 5:**
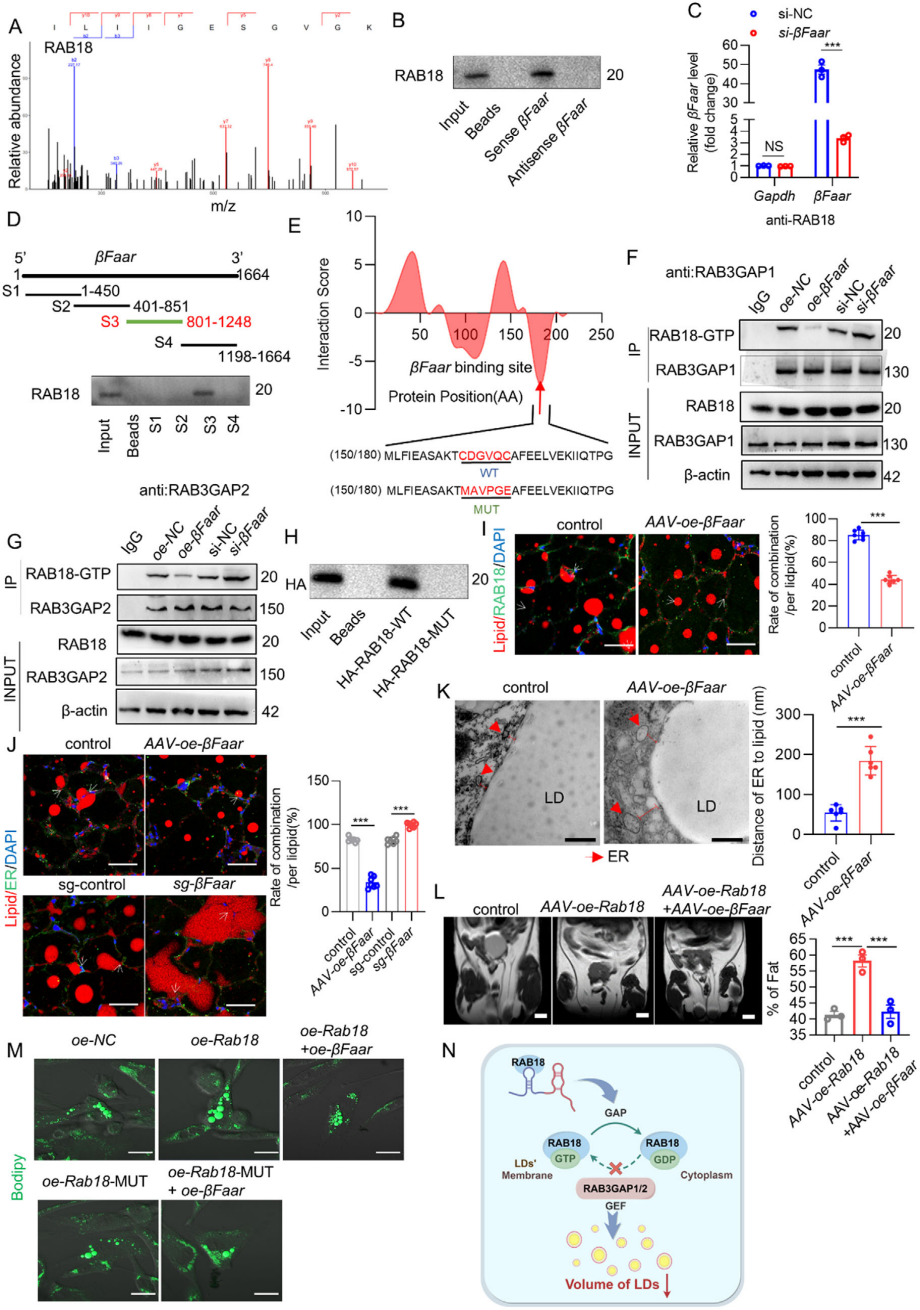
*βFaar* reduces LD‐ER apposition by binding RAB18, resulting in LD shrinkage. A) RNA pulldown assays were conducted using biotin‐labeled sense or antisense probes targeting *βFaar*, followed by mass spectrometry (MS) analysis of the retrieved RAB18 band. The corresponding peptide sequences are listed above the graphs. B) Immunoblotting analysis revealed representative images displaying the interaction between RAB18 and *βFaar* in the pulldown assay. C) Anti‐RAB18 RNA immunoprecipitation (RIP) was performed on 3T3‐L1 cell lysate transfected with si‐NC or *si‐βFaar*, and qRT‐PCR analysis revealed that precipitated RNAs contained RAB18–*βFaar* interaction, and *si‐βFaar* reduced the binding ability between RAB18 and *βFaar*. *Gapdh* was used as a control to validate this interaction. D) Mapping of RAB18‐binding domains of *βFaar* was carried out, including full‐length and truncated fragments of *βFaar*. Immunoblotting images showed RAB18 binding to different *βFaar* fragments in the RNA pulldown samples. E) Predicting *βFaar*‐RAB18 binding sites was done through catPARID databases (http://service.tartaglialab.com/). F,G) Co‐immunoprecipitation experiments with antibodies against RAB3GAP1 or RAB3GAP2 demonstrated that overexpression or knockdown of *βFaar* affected formal transformation of RAB18‐GTP in 3T3‐L1 cells transfected with *oe/sg‐βFaar* plasmid. H) Immunoblotting analysis revealed representative images displaying the binding site between RAB18 and *βFaar* in the pull‐down assay. I) Immunofluorescence of the interaction of RAB18 and LDs, as shown by representative images with a scale bar indicating 20 µm (*n* = 7 mice). J) Immunofluorescence of the interaction of ER and LDs, as shown by representative images with a scale bar indicating 20 µm (*n* = 7 mice). K) Representative electron microscopy (EM) images displayed the contact between ER and LDs in cells, indicated by red arrows pointing at ER cisternae structures (*n* = 6 mice). L) Representative coronal section MRI images and visceral and subcutaneous adipose tissue volume of HFD‐fed control, *AAV‐oe‐Rab18*, *AAV‐oe‐βFaar* and *AAV‐oe‐Rab18* & *AAV‐oe‐βFaar* mice (*n* = 3 mice). M) Representative images depicted LDs labeled green in *oe‐Rab18*, *oe‐Rab18‐*MUT, *oe‐Rab18*+oe‐*βFaar, or oe‐Rab18‐*MUT+*oe‐βFaar*‐transfected 3T3‐L1 preadipocytes, confirming their presence under different experimental conditions, with a scale bar indicating 20 µm. N) Schematic diagram showing the mechanism by which *βFaar* promotes RAB18‐GTP transform to RAB18‐GDP, inhibiting LDs mature. Schematic illustration was drawn by figdraw. The fold change in mRNA expression was calculated via the 2^−ΔΔCt^ method. The data are presented as the means ± SEMs. The *p* values obtained using a two‐tailed unpaired Student's *t*‐test or two‐way ANOVA are indicated; ***p < 0.001.

Mechanistically, we hypothesized that *βFaar* might bind to a protein that mediates its effects on LDs. To identify candidate proteins, we incubated in vitro transcribed biotinylated sense or antisense *βFaar* RNA with 3T3‐L1 cell lysates, performed pulldown with streptavidin beads and subjected the pulled‐down samples to mass spectrometry (MS) and western blot analyses (Figure S5A, Supporting Information). Silver staining revealed that more proteins were pulled down with the *βFaar* sense RNA than with the antisense RNA (Figure S5B, Supporting Information). The isolated proteins were then subjected to MS, revealing that a total of 347 proteins were pulled down by *βFaar*. Among them, RAB18 was one of the top ten candidate proteins identified (**Figure** **5**A). Since RAB18 is a well‐known master regulator of LD formation,^[^ ^17^ ^]^ we sought to establish a potential link between *βFaar* and RAB18. Western blotting of the pulled down proteins confirmed that RAB18 can bind to *βFaar* (Figure 5B). The interaction of *βFaar* with RAB18 was further validated by RNA immunoprecipitation (RIP) in 3T3‐L1 cells transfected with si‐NC or *si‐βFaar*. The RAB18 antibody successfully pulled down *βFaar*, and *si‐βFaar* reduced the binding ability between RAB18 and *βFaar*, as determined by qRT‒PCR (Figure 5C). Additionally, a series of truncated *βFaar* constructs were prepared to identify the specific region of *βFaar* that binds to RAB18. We found that a fragment at the 3′ end of *βFaar* (nt 801–1248) was sufficient for RAB18 binding (Figure 5D), while mutant region (nt 801–1248) of *βFaar* (*βFaar* MUT‐S3) almost have no binding ability with RAB18 (Figure S5C, Supporting Information). Furthermore, fluorescence in situ hybridization/immunofluorescence (FISH/IF) revealed that *βFaar* colocalized with RAB18 in the cytoplasm (Figure S5D, Supporting Information). These results confirmed the interaction between RAB18 and *βFaar*.

Consistent with previous reports,^[^ ^18^ ^]^ RAB18 expression levels were increased in the adipose tissue of obese mice (Figure S5E,F, Supporting Information). This prompted us to measure the RAB18 level in *βFaar*‐overexpressing 3T3‐L1 cells, but the RAB18 mRNA and protein levels did not change after *βFaar* was overexpressed (Figure S5G,H, Supporting Information), indicating that *βFaar* did not regulate the transcription or expression of RAB18. Since RAB18 is a small GTPase, we next investigated whether *βFaar* affects the activity of RAB18. Previous studies have shown that two cysteine residues at the carboxyl end of RAB18 can bind to the RAB3GAP1/2 complex, a specific GEF for RAB18, thereby activating RAB18.^[^ ^19^ ^]^ catRAPID database predictions indicated that *βFaar* may bind to the carboxyl end of RAB18 (Figure 5E). The coimmunoprecipitation (Co‐IP) results also revealed that *βFaar* overexpression significantly reduced the GTPase activity of RAB18 (Figure 5F,G). Finally, site‐directed mutagenesis was used to mutate the carboxyl terminal cysteine residue of RAB18 to alanine, which greatly reduced the binding strength between *βFaar* and the mutated RAB18 (Figure 5H).

Previous studies have demonstrated that LDs swell by acquiring neutral lipids from the ER and that RAB18 is a crucial regulator of vesicle trafficking between these two organelles.^[^ ^20^ ^]^ Therefore, we considered that *βFaar* could function in conjunction with RAB18 to modulate LD‐ER apposition. After inducing *βFaar* overexpression via in situ injection, we detected a decrease in the level of RAB18 on the surface of LDs, indicating that RAB18 was inactive (Figure 5I). Consistent with this finding, the contact between nascent LDs and the ER decreased in eWAT after *βFaar* was overexpressed, whereas the opposite effect was observed when *βFaar* was silenced (Figure 5J). Transmission electron microscopy observations confirmed this result (Figure 5K). We next explored the complementarity between RAB18 and *βFaar*. *βFaar* alleviated the fat accumulation induced by RAB18 and normalized the levels of related blood indicators (Figure 5L; Figure S5I, Supporting Information). Similarly, *βFaar* suppressed LD swelling caused by *Rab18*, while *βFaar* did not suppress LD swelling caused by overexpression a mutant of *Rab18* that lacks *βFaar* binding (oe‐Rab18‐MUT) in the 3T3‐L1 cells (Figure 5M). And 3T3‐L1 cells overexpressed *βFaar*‐MUT‐S3 cannot restrain LD swelling compared with control, *βFaar*‐MUT‐S3 also cannot suppress LD swelling caused by Rab18 in the 3T3‐L1 cells (Figure S5J, Supporting Information), TG content showed the same phenomenon (Figure S5K, Supporting Information). In addition, *βFaar* inhibited LD enlargement via its interaction with RAB18 in iWAT (Figure S5L, Supporting Information). However, Rab18 overexpression did not restore the *βFaar*‐mediated increase in UCP1 protein expression in iWAT (Figure S5M, Supporting Information). Taken together, these data suggest that the *βFaar*–RAB18 complex may be involved in the growth of LDs by reducing LD‐ER apposition in WAT (Figure 5N).

### 2.6 βFaar Regulates iWAT Browning by Promoting IRF4 Nuclear Translocation

**Figure 6:**
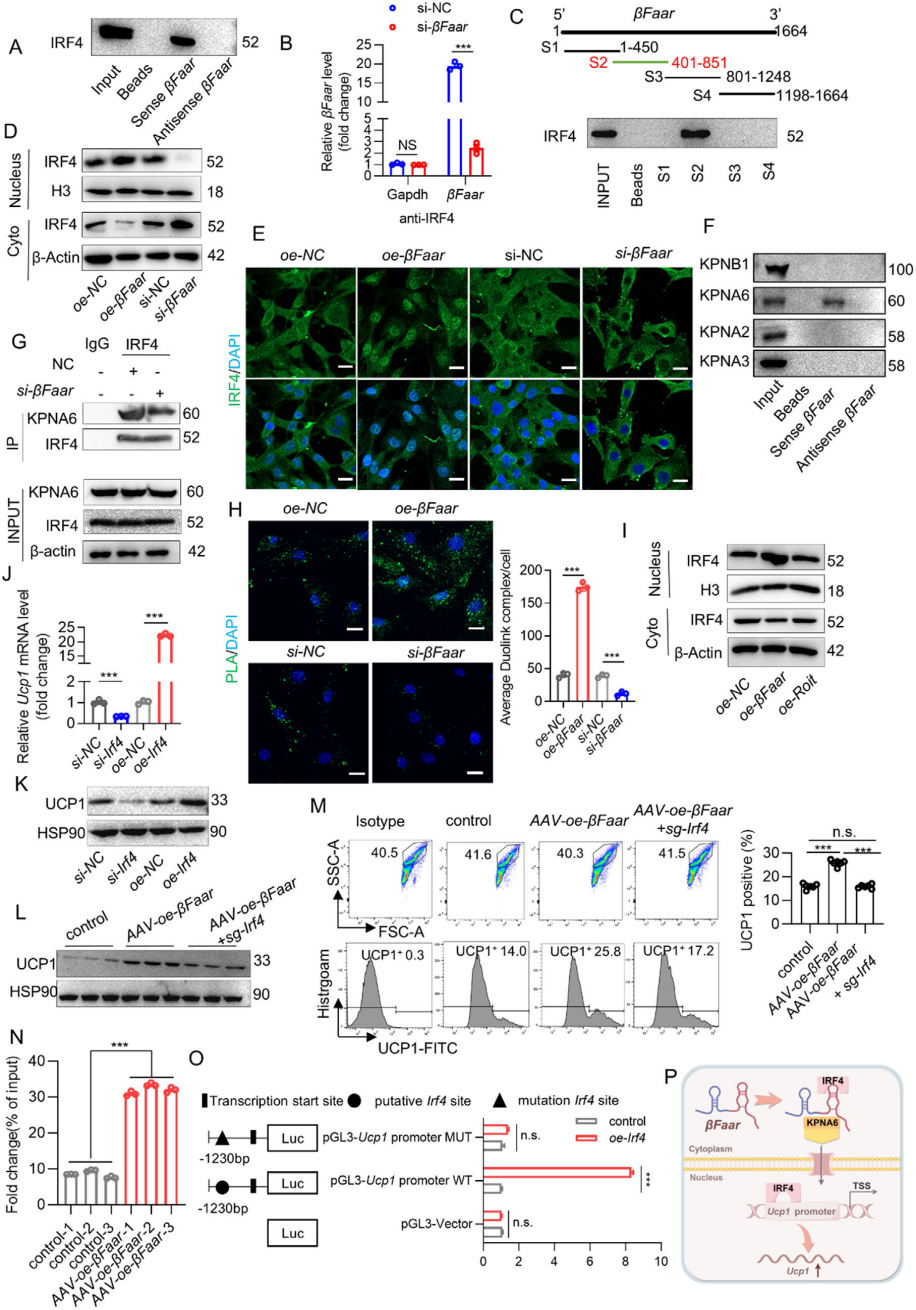
*βFaar* regulates WAT browning by promoting IRF4 nuclear translocation. A) Immunoblotting analysis revealed representative images displaying the interaction between IRF4 and *βFaar* in the pulldown assay. B) Anti‐IRF4 RNA immunoprecipitation (RIP) was performed on 3T3‐L1 cell lysate transfected with si‐NC or *si‐βFaar*, and qRT‐PCR analysis revealed that precipitated RNAs contained IRF4–*βFaar* interaction, and *si‐βFaar* reduced the binding ability between IRF4 and *βFaar*. *Gapdh* was used as a control to validate this interaction. C) Mapping of IRF4‐binding domains of *βFaar* (above), immunoblotting images showed IRF4 binding to different *βFaar* fragments in the RNA pulldown samples (bottom). D) Immunoblotting images depicted the subcellular distribution of IRF4 in *oe/si‐βFaar* 3T3‐L1 cells. E) Immunofluorescence analysis revealed the subcellular distribution of IRF4 in control and *oe/si‐βFaar* 3T3‐L1 cells, where green indicates IRF4 and blue indicates DAPI staining. With a scale bar of 40 µm. F) Immunoblotting confirmed that KPNA6 interacted with *βFaar* in the pull‐down assay. G) Immunoprecipitation of IRF4 by KPNA6 followed by immunoblotting analysis was conducted in control and *si‐βFaar* 3T3‐L1 cells. H) Representative images of the Duolink in situ PLA showing that there was a direct interaction between IRF4 and KPNA6, that the effect was enhanced after *βFaar* overexpression. I) Representative immunoblotting images demonstrated changes in both subcellular distribution and expression levels of IRF4 upon overexpression or knockdown of *βFaar* or *Roit* genes in 3T3‐L1 cells. J,K) Immunoblotting analysis and qRT‐PCR revealed the protein and mRNA levels of UCP1 were assessed in *oe/si‐Irf4* 3T3‐L1 cells to investigate their relationship with *Irf4* gene manipulation. L) Immunoblotting analysis revealed representative images displaying the UCP1 in *AAV‐oe‐βFaar* or *AAV‐oe‐βfaar+sg‐Irf4* mice (*n* = 3 mice). M) Flow cytometry assay was employed to detect the content of UCP1^+^ cells in iWAT after treating with *AAV‐oe‐βFaar*, *AAV‐oe‐βfaar+sg‐Irf4* mice or control mice (*n* = 7 mice). N) IRF4 could directly bind to *Ucp1* promoter, as evidenced by ChIP‐qPCR assays showing enrichment relative to IgG on the *Ucp1* promoter region when treated with *AAV‐oe‐βfaar* (*n* = 7 mice). O) A putative binding site for *Irf4* was identified within −1.5 kb upstream region from the primary transcript start site for *Ucp1* mutagenesis experiments targeting this binding site abolished *Irf4* induction activity observed in the original experiment using 3T3‐L1 cells. P) Schematic diagram showing the mechanism by which *βFaar* promotes the nuclear translocation of IRF4. Schematic illustration was drawn by figdraw. The fold change in mRNA expression was calculated via the 2^−ΔΔCt^ method. The data are presented as the means ± SEMs. The *p* values obtained using a two‐tailed unpaired Student's *t*‐test or two‐way ANOVA are indicated; ***p < 0.001.

RAB18 has no effect on iWAT browning; therefore, we next explored the mechanism underlying *βFaar*‐mediated iWAT browning. On the basis of the observed upregulation of UCP1 after *βFaar* overexpression, we hypothesized that *βFaar* may interact with other protein partners to promote iWAT browning. After reanalyzing the proteins pulled down by *βFaar*, we found that IRF4 also bound to *βFaar* (Figure S6A, Supporting Information). A previous study reported that IRF4 can promote the transcription of UCP1 in BAT,^[^ ^21^ ^]^ which prompted us to investigate the binding between *βFaar* and IRF4. RNA‒protein pulldown, RIP, and PCR assays confirmed that *βFaar* binds to IRF4 (**Figure** **6**A,B). A series of truncated *βFaar* constructs were prepared to identify the specific region of *βFaar* responsible for its binding to IRF4. We found that a fragment at the 3′ end of *βFaar* (nt 401–851), which was offset from the RAB18 binding site, was sufficient for IRF4 binding (Figure 6C), while mutant region (nt 401–851) of *βFaar* (*βFaar*‐MUT‐S2) almost have no binding ability with IRF4 (Figure S6B, Supporting Information). We next examined whether *βFaar* overexpression in 3T3‐L1 cells regulates IRF4 expression, and found that *βFaar* did not regulate IRF4 expression (Figure S6C,D, Supporting Information). FISH revealed that *βFaar* and IRF4 were present in both the cytoplasm and nucleus (Figure S6E, Supporting Information). Under cold stimulation, IRF4 was also translocated to the nucleus (Figure S6F, Supporting Information), where it induced the expression of UCP1. Thus, we investigated whether *βFaar* can regulate IRF4 nuclear translocation. The western blot data revealed that *βFaar* knockdown reduced the protein level of IRF4 in the nucleus, whereas *βFaar* overexpression had the opposite effect (Figure 6D). IF staining confirmed the regulatory effect of *βFaar* on IRF4 nuclear translocation (Figure 6E).

To understand the mechanism by which *βFaar* promotes IRF4 nuclear translocation, we again reanalyzed the proteins pulled down by *βFaar* and hypothesized that *βFaar* may bind to certain importins. As expected, several importins, such as KPNB1, KPNA2, KPNA3, and KPNA6, exhibited potential to bind with *βFaar* (Figure S6G–J, Supporting Information), and their interactions were subsequently investigated. Significant amounts of KPNA6, but not the other importins, were pulled down by the *βFaar* sense sequence (Figure 6F). Furthermore, the KPNA6 antibody precipitated a substantial amount of *βFaar* (Figure S6K, Supporting Information). We then sought to determine whether the interaction between *βFaar* and KPNA6 alters the binding of IRF4 to KPNA6. As shown in Figure 6G,H, both the Duolink in situ proximity ligation assay (PLA) and co‐IP results revealed that after deletion of *βFaar*, the interaction between KPNA6 and IRF4 decreased, indicating that *βFaar* facilitates the formation of the IRF4/KPNA6 complex. Consistent with the above results, *βFaar* overexpression increased the ability of KPNA6 to interact with IRF4 (Figure S6L, Supporting Information). Moreover, we confirmed that IRF4 is unable to bind directly to KPNA6 in the absence of *βFaar* through GST pulldown experiments (Figure S6M, Supporting Information). These results suggest that *βFaar* functions as a molecular scaffold that facilitates the interaction between IRF4 and KPNA6. Next, to determine whether the *βFaar*/KPNA6/IRF4 complex alters the nuclear translocation of IRF4, we transfected *βFaar*‐knockdown 3T3‐L1 cells with *βFaar* and an unrelated lncRNA (*Roit*)^[^ ^22^ ^]^ and utilized western blotting to analyze the distribution of IRF4 in the cytoplasm and nucleus. As shown in Figure 6I, transfection of *βFaar* restored the localization of IRF4 to the nucleus in *βFaar*‐knockdown 3T3‐L1 cells, whereas the lncRNA *Roit* did not. Collectively, these results support our hypothesis that the translocation of IRF4 from the cytoplasm to the nucleus depends on *βFaar*.

As mentioned above, *βFaar* overexpression leads to an increase in UCP1 expression, and high expression of UCP1 is a hallmark of WAT browning. Interestingly, the GAAA motif, an IRF4 target sequence, is present in the promoter of UCP1 (Figure S6N, Supporting Information), which prompted us to explore whether IRF4 can regulate the expression of UCP1 in iWAT. *Irf4* overexpression led to the significant upregulation of UCP1 at both the mRNA and protein levels, and *siRNA*‐mediated knockdown of *Irf4* had the opposite effect (Figure 6J,K). Moreover, UCP1 expression and UCP1^+^ cells were increased in the iWAT of *AAV‐oe‐βFaar*, while these increases were abolished in the iWAT of *AAV‐oe‐βFaar+AAV‐sgIrf4* mice compared to *AAV‐oe‐βFaar* mice (Figure 6L,M). While the UCP1 protein level and the mRNA levels of hallmark genes (including *Pgc1α* and *Cidea*) have no significant change in the 3T3‐L1 cells overexpressed *βFaar‐*MUT‐S2 compared with control, *βFaar‐*MUT S2 cannot restore UCP1 protein level and hallmark genes mRNA level decreased by *si‐Irf4* (Figure S6O,P, Supporting Information). Chromatin immunoprecipitation (ChIP) assays revealed that IRF4 directly bound to the promoter of UCP1 and that overexpressing *βFaar* increased this binding event (Figure 6N). We then used a dual‐luciferase assay to determine the level at which UCP1 transcription is regulated by IRF4. We constructed a luciferase reporter plasmid containing the ‐1500 to +1 (WT) portion of the UCP1 promoter region and mutant reporter plasmids with mutations from ‐1251 to ‐1241 (Mut). The overexpression of IRF4 significantly increased the luciferase activity of the WT but not the Mut in 3T3‐L1 cells (Figure 6O). Collectively, these results showed our hypothesis that the translocation of IRF4 from the cytoplasm to the nucleus depends on the *βFaar*/KPNA6 complex; furthermore, by promoting the nuclear transport of IRF4, a UCP1 transcription factor, *βFaar* regulated iWAT browning (Figure 6P).

### 2.7 In Vivo Overexpression of βFaar Induces WAT Browning and Protects Mice Against Obesity

**Figure 7:**
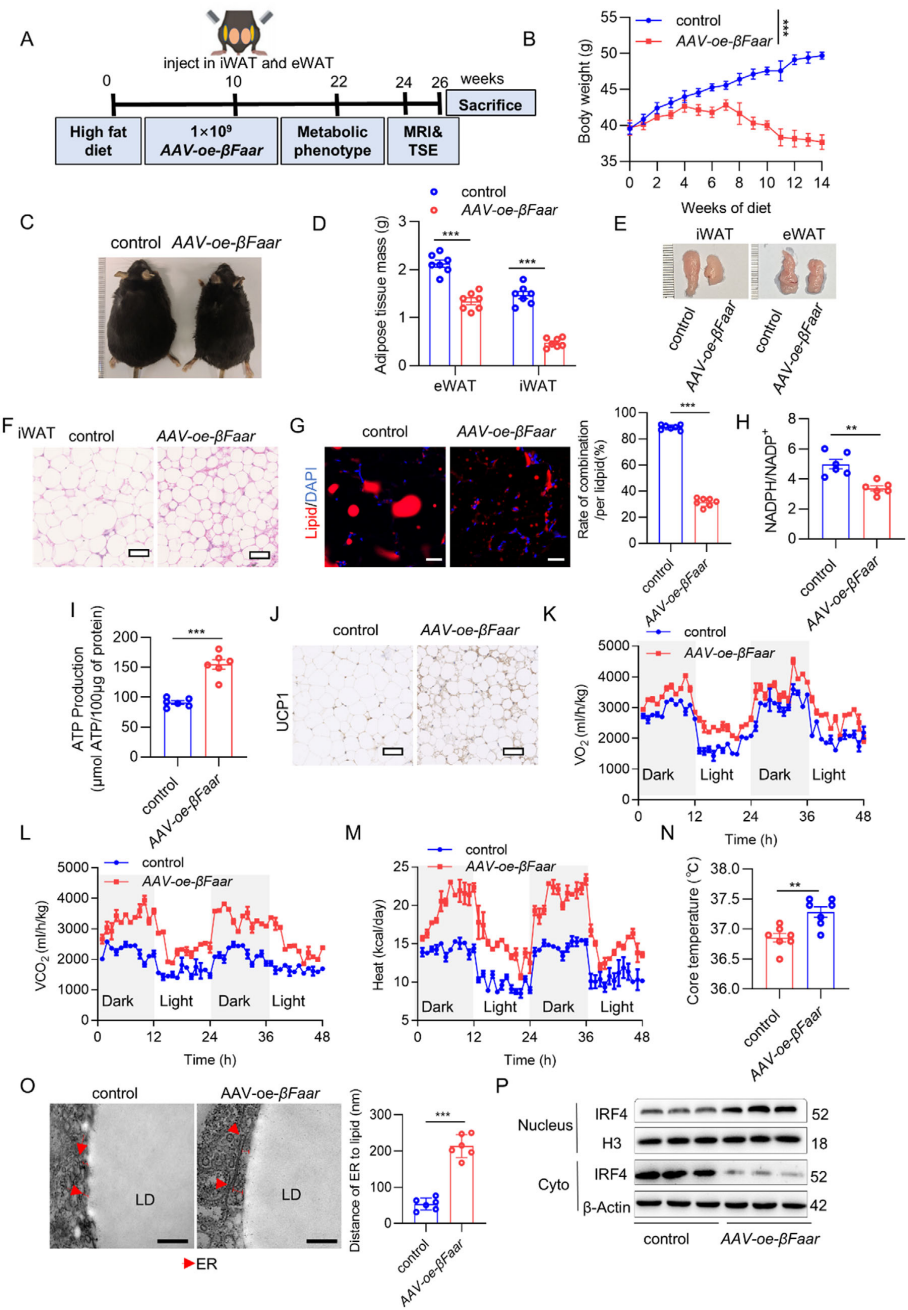
In vivo, overexpression of *βFaar* induces WAT browning and protects mice against obesity. A) Flowchart of the in vivo experiments to detect adipocyte function (*n* = 10 mice) in HFD‐fed mice treated with *AAV‐oe‐βFaar* and detection of the relevant indicators in the adipose tissue. B,C) The changes in body weight were measured for *AAV‐oe‐βFaar* HFD mice and control HFD mice separately (n = 7 mice). D,E) The changes in weight of eWAT and iWAT were measured for *AAV‐oe‐βFaar* mice (n = 7 mice). F) The images illustrating H&E staining in iWAT sections with a scale bar of 100 µm (*n* = 7 mice). Scale bar, 100 µm. G) The lipid immunofluorescence treated with *AAV‐oe‐βFaar* or *control* was obtained, with a scale bar of 20 µm (*n* = 7 mice). H,I) NADPH/NADP^+^ and ATP production were measured in iWAT treated with *AAV‐oe‐βFaar* (n = 6 mice). J) The images illustrating UCP1 immunohistochemistry in iWAT sections treated with *AAV‐oe‐βFaar* or control were captured with a scale bar of 100 µm (*n* = 7 mice). K–M) VO_2_ K), VCO_2_ L), and heat production M) analysis were performed using *AAV‐oe‐βFaar*. The day/night bar represents a 12‐hour duration. (*n* = 3 mice). VO_2_, VCO_2_, and heat production were analyzed by ANCOVA with total body mass as a covariate. N) Rectal temperature of mice was monitored after overexpression of *βFaar* (*n* = 6 mice). O) Representative EM images showed decreased contact between ER and LDs in *βFaar* overexpressing cells. ER are indicated with red arrows. P) Immunoblotting images depicted the subcellular distribution of IRF4 in iWAT of *AAV‐oe‐βFaar* mice (*n* = 6 mice). The fold change in mRNA expression was calculated via the 2^−ΔΔCt^ method. The data are presented as the means ± SEMs. The *p* values obtained using a two‐tailed unpaired Student's *t*‐test or two‐way ANOVA are indicated; ***p < 0.001.

To prove the therapeutic potential of *βFaar*, we administered *βFaar* to mice with HFD‐induced obesity. Eight‐week‐old male C57BL/6J mice were fed an HFD for 10 weeks, and then their iWAT and eWAT were injected in situ with *βFaar*, followed by continuous observation for 16 weeks (**Figure** **7**A). The AAV8‐*Adipoq* promoter‐pHAGE ZsGreen‐CMV vector used also expressed GFP to monitor viral infection in tissues. Local injection of *AAV‐oe‐βFaar* into iWAT and eWAT resulted in effective adipose tissue infection (Figure S7A, Supporting Information). Consistent with the high infection efficacy, the *βFaar* levels in iWAT and eWAT increased by more than 8‐fold (Figure S7B,C, Supporting Information). To determine the overall phenotype of mice with WAT overexpression of *βFaar*, body weight, WAT mass, food intake, and glucose and insulin tolerance were evaluated (Figure 7B–E; Figure S7D–F, Supporting Information). Detailed analysis of the adipose depots revealed that *βFaar* treatment induced a significant phenotypical change in the iWAT, as indicated by decreased adipocyte size (Figure 7F), whereas the LD size in eWAT decreased in response to increased *βFaar* expression (Figure 7G). In addition to the phenotypic changes observed in the adipocytes of the iWAT, the iWAT also exhibited increased mitochondrial function (Figure 7H,I). Furthermore, the UCP1 protein was detectable in the iWAT of *βFaar*‐overexpressing mice, indicating browning and thermogenic activity (Figure 7J).

In addition, oxygen consumption by and the metabolic rate and core body temperature of the mice were measured to determine whether the overexpression of *βFaar* in WAT influences total energy expenditure. Upon cold exposure (22 °C), *βFaar* overexpression led to a significant increase in the metabolic rate (Figure 7K–M) and an increase in the core body temperature (Figure 7N). Consistent with these phenotypic changes, delivery of *βFaar* to mice robustly reduced the contact between the LDs and ER in eWAT (Figure 7O). Additionally, significant increases in IRF4 nuclear translocation were detected in the iWAT of *βFaar*‐treated mice (Figure 7P). We also examined whether the overexpression of *βFaar* in WAT leads to changes in BAT via immunohistochemical analysis of adipocyte morphology and quantification of UCP1 expression in *βFaar‐*treated mice. However, unlike WAT, systemic delivery of *βFaar* had no significant effect on BAT (Figure S7G, Supporting Information). These findings further confirm that one of the functions of *βFaar* is the browning of iWAT.

## 3 Discussion

**Figure 8:**
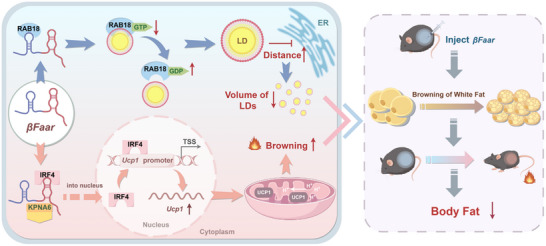
Schematic of the mechanism by which *βFaar* reduces body fat by promoting white adipose tissue browning and inhibiting LD enlargement. In obese mice, *βFaar* selectively targets and inhibits the GTPase activity of the RAB18 protein, thereby reducing LD volume. Conversely, *βFaar* promotes nuclear translocation of the transcription factor IRF4 in inguinal white adipose tissue (iWAT), facilitating the browning of white adipose tissue and attenuating body fat accumulation. Schematic illustration was drawn by figdraw.

Given the increasing prevalence of obesity, there is an urgent need to develop new, effective strategies for its treatment. One promising approach is the induction of WAT browning. Our initial studies revealed that *βFaar* was downregulated in the adipocytes of mice and humans with obesity.^[^ ^16^ ^]^ In this study, we report that *βFaar* overexpression in adipose tissue inhibited the growth of LDs, accelerated iWAT browning and thermogenesis, and increased energy expenditure to protect against HFD‐induced obesity and metabolic dysregulation. Mechanistically, our work provides the first evidence that *βFaar* interacts with RAB18 inhibition to expand LDs by reducing the distance between LDs and the ER; on the other hand, *βFaar* interacts with KPNA6 and promotes IRF4 nuclear translocation to increase UCP1 transcription, thus accelerating iWAT browning and increasing energy expenditure. Our findings revealed the pivotal role of *βFaar* in orchestrating WAT expansion and promoting browning (**Figure** **8**).

With the development of sequencing technology, many lncRNAs have been discovered in adipocytes,^[^ ^23^, ^24^, ^25^ ^]^ but our understanding of their biological functions is limited. Our work revealed that *βFaar* was highly expressed in iWAT and eWAT, upregulated upon cold exposure, and downregulated in obese individuals. Increased WAT *βFaar* expression may serve as a checkpoint in the conversion of WAT to BAT. Several findings support this model. First, the upregulation of *βFaar* in WAT can increase the basal metabolic rate and body temperature of mice. Furthermore, *βFaar* deficiency in mice failed to fully activate the WAT browning program following the activation of β3‐adrenergic signaling. Importantly, adipocyte‐specific overexpression of *βFaar* can directly promote browning of adipose tissue in obese mice. *βFaar* overexpression enhanced the morphological and molecular hallmarks associated with browning of white adipocytes, whereas knocking down *βFaar* elicited the opposite effect. These results illustrate that increased expression of *βFaar* in adipose tissue serves as an adaptive mechanism to promote healthy adipose tissue remodeling and maintain metabolic homeostasis in individuals with obesity. Moreover, previous studies have shown that the promotion of WAT browning is related to temperature.^[^ ^26^, ^27^ ^]^ However, *βFaar* promotes WAT browning in a temperature‐independent manner. These findings suggest that the mechanism by which *βFaar* promotes the browning of WAT is unique.

The mechanisms through which *βFaar* promotes WAT browning and prevents diet‐induced obesity are likely multifactorial. First, *βFaar* inhibits the GTPase activity of RAB18, thereby hindering the LD‐ER apposition, resulting in smaller and less mature LDs. The dynamics of LDs reflect lipid metabolic status, and uncontrolled LD growth has been linked to the initiation of obesity LD biogenesis^[^ ^28^ ^]^; furthermore, nascent LDs are formed in the ER.^[^ ^29^, ^30^ ^]^ Nascent LDs may become mature LDs by acquiring neutral lipids from the ER through their continuous association with this organelle.^[^ ^31^ ^]^ RAB18 can promote LD growth by tethering the LDs to the ER through the interactions of SNARE and NRZ.^[^ ^20^ ^]^ Analysis of the MS and Co‐IP data revealed that *βFaar* can bind to the GTPase RAB18, and *βFaar* overexpression significantly reduced the content of RAB18. Electron microscopy confirmed that *βFaar* hindered the binding of RAB18‐mediated LDs to the ER. These findings explain the decrease in the number of adipocytes observed after *βFaar* is overexpressed.

Previous studies have revealed that some lncRNAs regulate key proteins including HIF1α,^[^ ^32^ ^]^ Prdm16,^[^ ^33^ ^]^ UCP1,^[^ ^34^, ^35^ ^]^ hnRNP‐U,^[^ ^23^ ^]^ etc.,^[^ ^36^ ^]^ Adiponectin,^[^ ^37^ ^]^ Zbtb7b,^[^ ^38^ ^]^ Klf12,^[^ ^39^ ^]^ Dio3o,^[^ ^40^ ^]^ Pka,^[^ ^41^ ^]^ Fabp4,^[^ ^42^ ^]^ Sirt1^[^ ^43^ ^]^ that affect WAT browning. UCP1 is a unique protein that is expressed in the inner mitochondrial membrane of brown and beige adipocytes and uncouples oxidative phosphorylation from ATP synthesis to release energy in the form of heat.^[^ ^4^, ^44^ ^]^ Though, our results showed that *βFaar* overexpression induced UCP1 expression and WAT browning to drive energy consumption in HFD‐fed mice, Co‐IP–MS analysis showed that *βFaar* cannot directly bind to UCP1. Notably, the present study revealed that the IRF4 protein binds to *βFaar* to promote the transcription of UCP1 in BAT.^[^ ^21^ ^]^ More Interesting, our results demonstrated that *βFaar* does not affect IRF4 transcription or translation; instead, we revealed a new phenomenon in which IRF4 is translocated to the nucleus as part of the *βFaar*/IRF4/KPNA6 complex and binds to the promoter of UCP1 in a manner dependent on its interaction with *βFaar*. By enhancing the transcriptional activity of UCP1, the *βFaar*/IRF4/KPNA6 complex induces WAT browning. When the interaction between *βFaar* and KPNA6 was interrupted by mutation of the binding site, the translocation of IRF4 decreased, suppressing WAT browning. This finding suggests a model of cotransport for *βFaar*/KPNA6 and UCP1‐associated transcription factors such as IRF4, which is unique to *βFaar*.

Obesity is the result of the accumulation of excess energy in WAT, whereas BAT, which specializes in energy expenditure through thermogenesis, potently counteracts obesity. Factors that induce brown adipocyte commitment and energy expenditure are likely promising targets for treatments against adiposity. Here, we show that *βFaar*‐overexpressing mice exhibit a striking increase in energy expenditure and BAT‐like adipocytes in WAT depots with increased expression of BAT markers. Our results revealed the important roles of *βFaar* in energy balance and body weight control through its regulation of the white‐to‐brown fat transition. However, the most significant issue for the clinical application of *βFaar* is its efficient delivery to the target site. Thus, further studies are necessary to design appropriate delivery methods or construct delivery vectors, such as via chemical modification or the fabrication of lipid‐based or polymer‐based nanocarriers, to transport *βFaar* to adipose tissue to fully exploit its great potential for obesity treatment.

## 4 Conclusion

In summary, we demonstrated the protective role of *βFaar* in iWAT and eWAT during obese development. We here propose two critical axes: the *βFaar*–Rab18 axis in control of LD swelling, and 2) the *βFaar*–KPNA6–IRF4 axis promoted IRF4 nuclear translocation, increasing UCP1 transcription, which finally increased iWAT browning. Our experiments suggested that *βFaar* may be a potential therapeutic target for the management of obesity and related disorders.

## 5 Experimental Section

### Study Approval

The adipose tissue and clinicopathological data were collected at Sir Run Run Hospital, Nanjing Medical University (Nanjing, China). All participants in this study were classified as obese (BMI > 25), whereas the control group consisted of individuals of normal weight (20 ≤ BMI ≤ 25). Informed consent was obtained from all the human subjects. The research involving human subjects adhered to the principles outlined in the Declaration of Helsinki and received approval from the Ethics Committees of the Department Sir Run Run Hospital (Nanjing, China; ethical code: 2023‐SR‐046). Table S1 (Supporting Information) provides an overview of the clinical characteristics of the patients.

### Animal Studies

The animals were cared for according to the guidelines set by the institutional animal care committee. All procedures were approved by the animal ethics committee of China Pharmaceutical University (permit number: 2 162 326) and complied with international laws and policies (EEC Council Directive 86/609, 1987). The mice used in this study were primarily on the C57BL/6J background, except for the *ob/‐* mice and *ob/ob* mice, which were on the BKS background. All the mice were housed in a specific pathogen‐free facility in a temperature (22 °C) and humidity (55%) controlled room on a 12 h light‐dark cycle (lights on from 6 a.m. to 6 p.m.). Unless specified otherwise, the animals were fed an NCD (D12450J) that provided ≈10% calories from fat and had ad libitum access to water. To establish a model of diet‐induced obesity, the animals were fed an HFD (D12494) that provided ≈60% calories from fat for at least eight weeks. The ages of the mice were indicated in the figures; if no age was given, the mice were more than eight weeks of age. All the mice used in the experiments were male.

### 3T3‐L1 Cell Culture and Differentiation

3T3‐L1 cells were cultured in high‐glucose Dulbecco's modified Eagle's medium (DMEM; Gibco) supplemented with 10% calf serum at 37 °C with 5% CO_2_ and saturated humidity. They grew until they reached ≈80–90% confluence, at which time the culture medium was replaced with initial differentiation medium (high‐glucose DMEM, 10% fetal bovine serum (FBS), isobutylmethylxanthine (IBMX) (0.5 mm), dexamethasone (1 µm), and insulin (10 µg mL^−1^)). After 48 h of incubation, the media was replaced with the final differentiation cocktail comprising high‐glucose DMEM, 10% FBS, and insulin (10 µg mL^−1^) for an additional 48 h of incubation.

### Adipocyte Sample Preparation and SVF Differentiation

The SVF and mature adipocytes were obtained by digesting adipose tissue samples with collagenase type 1 in KRH buffer at 37 °C for 30 min. The resulting suspensions, which contained both mature adipocytes and SVF, were then filtered through nylon mesh and washed three times with KRH buffer, after which the mature adipocytes floated on the surface of the suspension, and the SVF was obtained by centrifuging the remainder of the mixture at 1500 rpm for 5 min. The resulting pellet was washed with preadipocyte growth medium (DMEM‐F12 supplemented with 10% calf serum and 1% penicillin‒streptomycin) and centrifuged again. The SVF cells were cryopreserved in freezing medium (DMEM‐F12 supplemented with 90% FBS and 10% dimethyl sulfoxide (DMSO)) by gradually reducing the temperature of the suspension at a rate of −1 °C min^−1^ before storage in liquid nitrogen until analysis. The whole adipose tissue samples collected during this study were rapidly frozen in liquid nitrogen and stored until analysis.

The SVF cells were cultured in DMEM‐F12 at 37 °C with 5% CO_2_ and saturated humidity. The cells grew until they reached ≈80%‐90% confluence, at which time the culture medium was replaced with an initial differentiation medium (10% FBS, IBMX (0.5 mm), dexamethasone (1 µm), and insulin (10 µg mL^−1^)). After incubation for 48 h, the media was exchanged for the final differentiation cocktail comprising 10% FBS and insulin (10 µg mL^−1^) for an additional 48 h of incubation.

### RNA Isolation and qRT‐PCR Analysis

RNA was extracted from WAT, BAT, muscle, heart, kidney, and brain tissues and 3T3‐L1 cells using TRIzol reagent (Invitrogen). Then, cDNA was synthesized via qRT‐PCR (LightCycler 480, Roche) from 500 ng of total RNA using the PrimeScriptTM RT reagent Kit (Takara, Tokyo, Japan) to measure mRNA expression levels. The relative expression levels of the target genes were determined using the 2^−ΔΔCT^ method after normalization to *Gapdh* expression. The sequences of primers used in this study are given in Table S2 (Supporting Information).

### Subcellular Fractionation

3T3‐L1 cells (1 × 10^6^ cells) were incubated for 5 min in Cell Fractionation Buffer at low temperature (PARIS Kit, Life) and then centrifuged at 500× g for 5 min, and the supernatant (cytoplasmic fraction) was collected. Additionally, the resulting pellet was suspended in Cell Disruption Buffer at low temperature (4 °C) for 10 min before centrifugation at 500× g for 5 min, to yield the pellet (nucleoplasmic fraction).

### Western Blot Analysis

Proteins were extracted from tissues or cells in radioimmunoprecipitation assay (RIPA) buffer (Beyotime) containing a complete protease inhibitor cocktail (Roche), resolved by SDS‒PAGE, transferred to polyvinylidene fluoride (PVDF) membranes (Bio‐Rad), and then probed with primary antibodies against RAB18 (#ab224466), UCP1 (#ab209483) (Abcam); IRF4 (#4964), HA‐tag (#3724), and β‐Actin (#4967) (CST); and RAB3GAP1 (#A18587), RAB3GAP2 (#ab17669), KPNA2 (#A5012), KPNA3 (#A16907), KPNA6 (#7363), KPNB1 (#A23235), FABP4 (#A11481), C/EBPα (#A25033), HSP90 (#A5027) and Histone H3 (#A2348) (ABclonal). The protein bands were visualized with enhanced chemiluminescence reagents (GE Healthcare) and quantified using ImageJ software.

### Flow Cytometric Analysis of UCP1^+^ Cells

Mature adipocytes were suspended in 1 mL of Live/Dead Fixable Dead Cell Stain (Molecular Probes) and incubated at low temperature for 30 min. Subsequently, the cells were washed once with FACS buffer (1% bovine serum albumin (BSA) in 1× phosphate‐buffered saline (PBS)) and treated with various antibodies. To analyze UCP1 positive cells, first, Freshly isolated cells (100 000) were subjected to triple staining with UCP1 (#MAB6158‐SP, R&D Systems; diluted at 1:100) or the respective isotype controls (BioLegend) in the dark on ice for 24 h. Next, FITC‐conjugated goat anti‐mouse IgG (H+L) (#AS001, ABclonal; diluted 1:1000) was applied, and after staining, the cells were fixed in 2% (w/v) paraformaldehyde and stored at 4 °C until analysis with a FACSCelesta Cell Analyzer (BD Biosciences).

### Plasmid and shRNA Construction

The coding sequences for *Rab18* (NC_000084.7) and *Irf4* (NC_000079.7) were amplified by PCR from full‐length mouse cDNA and then cloned and inserted into the *Adipoq* promoter‐pHAGE‐CMV vector; all the plasmids were confirmed by sequencing. The sequences of the primers used for PCR are listed in Table S3 (Supporting Information).

The shRNAs of *Rab18* and *Irf4* were constructed in the plvx‐shRNA2 lentiviral vector (Takara) with *EcoRI* and *BamHI* restriction sites. The shRNA sequences are listed in Table S4 (Supporting Information).

### Transfection of the lncRNA Smart Silencer

The lncRNA smart silencer (synthesized by RiboBio, Guangzhou, China) consisted of three siRNAs and three antisense oligonucleotides (ASOs) for targeted knockdown of *βFaar* in both the cytoplasm and nucleus; these sequences used can be found in Table S5 (Supporting Information). For transient transfection, ≈5 ×10^5^ 3T3‐L1 cells were seeded in six‐well plates, cultured without antibiotics, and transfected with the *βFaar* silencer or control *si‐NC* (each at a final concentration of 50 nmol L^−1^) using Lipofectamine 2000 (Invitrogen) following the manufacturer's instructions. After 24 h, the media was exchanged for fresh media containing antibiotics. At 48 h posttransfection, the cells were lysed, and total RNA or protein was extracted to assess knockdown efficiency.

### Administration of the AAV Vectors

AAV‐Cre administration was performed as previously described.^[^ ^45^ ^]^ The AAV8‐*Adipoq* promoter‐CMV‐ZsGreen‐*βFaar* (*AAV‐oe‐βFaar*) or AAV8‐*Adipoq* promoter‐CMV‐ZsGreen‐*Rab18 (AAV‐oe‐Rab18)* was used for WAT overexpression mice, whereas AAV8‐*Adipoq* promoter‐CMV‐ZsGreen‐pHAGE was used for oe‐control group. AAV8‐Adipoq promoter‐ pCas‐Puro‐U6*‐sgβFaar* (*sg‐βFaar*) or AAV8‐Adipoq promoter‐pCas‐Puro‐U6‐*sgIrf4* (*sg‐Irf4*) was used for WAT knockdown mice Mouse, and pCas‐Puro‐U6 was used for sg‐control group. Mouse WAT pads on both sides were injected. The mice were first anesthetized with isoflurane gas. Then, longitudinal incisions were made on the skin around the inguinal areas and epididymal region on both sides, and tweezers were used to expose the fat pads. Finally, *AAV‐oe‐βFaar*, *AAV‐oe‐Rab18*, *sg‐βFaar*, *sg‐Irf4* or control vector (1×10^10^ viral genomes (vg) in 100 µl) was injected into multiple locations (5–8) of each fat pad.

### Metabolic Studies in Mice

After 12 h of fasting, the blood glucose levels of the mice were measured using a glucometer (OMRON, Japan), whereas the fasting serum insulin levels were assessed using an insulin enzyme‐linked immunosorbent assay (ELISA) kit (Crystal Chem, USA)) following the manufacturer's instructions. For the GTTs, 2 g kg^−1^ glucose (Sigma‒Aldrich, St. Louis, MO, USA) was injected intraperitoneally into the mice, whereas the mice were injected intraperitoneally with 0.75 U kg^−1^ Novolin R insulin (Novo Nordisk, Bagsvaerd, Denmark) for the ITTs. Blood glucose levels were monitored at 0, 15, 30, 60, 90, and 120 min following the administration of either glucose or insulin. Serum samples were collected via eye canthus blood collection at 0, 5, 15, and 30 min after glucose injection. The incremental area under the curve (AUC) was calculated utilizing the conventional trapezoid rule.

### Body Composition Analysis

The changes in body composition were assessed as previously described.^[^ ^46^ ^]^ In brief, the mice were anesthetized with a 2% isoflurane solution and securely positioned on an MRI platform (Bruker BioSpec 7T/20 USR), after which anesthesia was consistently maintained with 1% isoflurane. Then, MRI images of the cross‐sections of the internal adipose tissue were obtained via sequential scanning. Statistical analysis of the lipid distribution in the mice was performed on the basis of the images using ImageJ software.

### Energy Expenditure

Mice were individually placed in the respiratory chambers of a TSE PhenoMaster/LabMaster (Germany) to monitor the 24 h respiratory parameters after drug or distilled water administration on days 20 and 30 at 8:00 a.m. The mice were continuously recorded for 24 h, and measurements of water intake, food intake, and gas exchange (O_2_ and CO_2_) (using the TSE LabMaster system) were taken every 30 min. Then, VO_2_, VCO_2_, and energy expenditure (EE) were calculated according to the manufacturer's guidelines (PhenoMaster Software, TSE Systems). The respiratory exchange rate was estimated by calculating the VCO_2_/VO_2_ ratio (RER), and the values were adjusted on the basis of body weight to the power of 0.75 (kg^−0.75^) where noted. The mice were placed in an isolated environment where the temperature was controlled at 22 ± 1 °C. The sampling flow rate was 0.25 L min^−1^ and the flow speed was 0.35 L min^−1^.

### Core Temperature, Thermal Imaging, and Cold Exposure

Heat production was evaluated using a thermo‐monitor system (Columbus Instruments), and measurements of each chamber were taken every 20 min. Infrared technology (Columbus Instruments) was utilized to quantify physical activity. The rectal core temperature was measured using a thermometer, whereas an infrared thermal camera (T1010, FLIR) was used to capture thermal images. To induce cold exposure, the mice fed an HFD were individually placed in plastic cages at 4 °C for 12 h. Core body temperature was measured post‐dissection with a thermometer.

### Immunohistochemistry (IHC)

The liver, eWAT, and iWAT were fixed with 4% paraformaldehyde, embedded in paraffin, and sectioned into 7 µm slices. The sections were subsequently deparaffinized, rehydrated, and subjected to antigen retrieval. To inhibit endogenous peroxidases, the tissue sections were treated with 3% H_2_O_2_ in methanol. Permeabilization was achieved by adding 0.1% Triton X‐100, followed by blocking with 5% BSA. The membranes were incubated with primary antibody overnight at 4 °C, washed with PBS, and exposed to horseradish peroxidase (HRP)‐conjugated goat anti‐rabbit IgG. Finally, detection was performed using a 3,3′‐diaminobenzidine (DAB) substrate kit.

### Measurement of Serum TG and FFA Levels

The serum levels of total TGs and FFAs were determined with an automated biochemical analyzer (Hitachi 7600, Tokyo, Japan) and TG or FFA reagents (Solarbio Life Sciences).

### Measurement of Muscle CK and TG Levels

Take 50 mg muscle tissue and obtain tissue solution by grinding. Following centrifugation at 10 000× g for 5 min, the upper organic phase was collected, the muscle levels of CK and TG were determined with an automated biochemical analyzer (Hitachi 7600, Tokyo, Japan) and CK or TG reagents (Solarbio Life Sciences).

### FISH

The Cy3‐labeled βFaar probe was synthesized and designed by GenePharma (Shanghai, China). For the FISH assay, 1×10^5^ 3T3‐L1 cells were fixed in 4% formaldehyde, permeabilized with 0.3% Triton X‐100 for 15 min, and washed three times with PBS and once with 2× SSC buffer. Hybridization was performed at 37 °C for 16 h using DNA probe sets before observation either directly or via IF, as described below. The signals were detected using a laser scanning confocal microscope (Zeiss LSM 800) with a 40× objective.

### IF

The eWAT and iWAT samples were fixed with 4% paraformaldehyde, and embedded in paraffin, after which antigen retrieval was performed by boiling in 10 mmol L^−1^ Tris/EDTA buffer (pH of 9.0). Then, permeabilization and blocking were performed in PBS containing 0.3% Triton X‐100, 1% BSA, and 5% goat serum. The primary antibody was then added for incubation overnight at 4 °C, after which the secondary antibody was added for one hour of incubation at room temperature. The signals were detected using a laser scanning confocal microscope (Zeiss LSM 800) with a 20× or 40× objective.

### Measurement of the Cellular TG and NEFA Contents

Differentiated 3T3‐L1 cells were gently removed from the culture dishes, washed with PBS, and lysed in lipid extraction solution (3:2 hexane:isopropanol, v/v). Following centrifugation at 1000× g for 5 min, the upper organic phase was collected, and the solvent was removed under nitrogen gas. The remaining lipids were reconstituted in chloroform supplemented with 1% Triton X‐100, and then the solvent was removed under nitrogen gas again. Finally, the lipids were suspended in water, and the TG levels were determined using TG reagent (Solarbio Life Sciences) and the NEFA levels were measured using NEFA reagent (Sigma‒Aldrich).

### Oil Red O Staining

Fully differentiated adipocytes were washed with PBS, fixed with 4% paraformaldehyde, incubated with 0.5% (w/v) Oil red O solution (in 60% isopropanol) for 1 h, and washed several times with water. The signals were detected via microscopy with a 20× objective.

### Immunostaining for LDs and the ER

3T3‐L1 cells or SVF cells that had undergone differentiation were prepared for immunostaining by being cultured on coverslips and then washed twice with PBS. The samples were subsequently fixed with a 4% paraformaldehyde solution for 20 min, followed by permeabilization with 0.1% saponin for the same duration. To visualize the LDs, the cells were stained with BODIPY 493/503 (1:200 dilution in PBS) for 20 min, while the ER was stained with ER‐Tracker Green at the same dilution in PBS for an additional 30 min. The signals were detected using a laser scanning confocal microscope (Zeiss LSM 800) with a 40× objective.

### Mitochondrial Function and Respiration

A Seahorse XFe96 Analyzer (Agilent) was used for to measure the mitochondrial OCRs in adipocytes according to the instructions of the Cell Mito Stress Test Kit (Agilent, cat. no. 103015–100). Briefly, SVF cells from eWAT were seeded onto an XFe96 cell culture microplate (Agilent) and differentiated into mature adipocytes. After 1 h of equilibration in XF DMEM at 37 °C without CO_2_, the XFe96 plate was transferred to the Seahorse XFe96 analyzer for data collection. The sequential addition of oligomycin (final concentration: 1.5 µm), carbonyl cyanide 4‐(trifluoromethoxy) phenylhydrazone (FCCP; final concentration: 2 µm), and rotenone/actinomycin A (final concentration: 0.5 µm each) into the microplate was performed using automatic pneumatic injection, followed by OCR measurement. Total protein from each well was subsequently extracted and quantified from the post‐OCR measurement. Data analysis was conducted using Seahorse Wave Desktop software (Agilent).

### Transmission Electron Microscopy

Mouse eWAT was dissected into fragments (≈1–2 mm each), which were immersed in 5% glutaraldehyde fixative solution for two days, followed by postfixation with 1% osmium tetroxide. To optimize visualization, the samples were stained with a 2% aqueous uranyl acetate solution for two hours before being dehydrated through graded ethanol solutions up to 100%. Subsequently, the samples were embedded in epoxy resin, made into ultrathin sections using an EM UC7 ultramicrotome (Leica), subjected to lead nitrate poststaining treatment, mounted on Formvar‐coated nickel grids, and examined under an FEI Tecnai G2 electron microscope.

### RNA‐Protein Pulldown

First, in vitro translation assays were performed using a T7 RNA polymerase transcription kit according to the manufacturer's instructions (Thermo, MA, USA). Total RNA was treated with RNase‐free DNase I (Roche, Basel, Switzerland) and then purified with an RNeasy Mini Kit (QIAGEN). *βFaar* RNAs, truncated fragments of *βFaar*, *βFaar*‐MUT‐S2 or *βFaar*‐MUT‐S3 were subsequently labeled using the Pierce RNA 3′ End Desthiobiotinylation Kit (Thermo). Biotinylated RNAs were incubated with the cytoplasmic extract of 3T3‐L1 cells (5 × 10^6^) at 4 °C for 2 h. Washed streptavidin agarose beads (Invitrogen) were added to each reaction mixture for incubation at room temperature for 1 h. The recovered proteins associated with *βFaar* or the control were resolved by gel electrophoresis and Coomassie brilliant blue staining. The eluted solutions were subjected to MS analysis (Shanghai Applied Protein Technology Co., Ltd.) on a Q Exactive mass spectrometer (Proxeon Biosystems, Thermo Fisher Scientific) or Western blotting.

### RIP

An EZMagna RIP kit (Millipore, Billerica, MA, USA) was used for RIP following the manufacturer's instructions. 3T3‐L1 cells were lysed with complete RIP lysis buffer, and 100 µL of the whole‐cell extract was incubated with RIP buffer containing magnetic beads conjugated with specific antibodies against RAB18 (Abcam, #ab224466), IRF4 (CST, #4964), or KPNA6 (ABclonal, #7363); normal mouse IgG (Abcam, #ab172730) was used as a negative control. Proteinase K treatment and subsequent isolation of the immunoprecipitated RNA were performed. The RNA concentration was determined using a microplate reader (Synergy 2, BioTek, USA), whereas the RNA quality was assessed using a bioanalyzer (Agilent, Santa Clara, CA, USA). Additionally, the purified RNA was subjected to qRT‐PCR analysis with specific primers targeting *βFaar* to confirm the presence of binding targets.

### Co‐IP Assays

After overnight incubation at 4 °C with the specified antibodies or IgG (CST, 3900 and 5415), protein samples were treated with protein A/G magnetic beads (MedChemExpress, HY‐K0202), and the mixture was incubated on a rotator for an additional 2 h. Subsequently, the immunoprecipitates were centrifuged and washed six times with wash buffer containing 150 mmol L^−1^ NaCl, 20 mmol L^−1^ Tris‐HCl (pH 7.5), 5% glycerol, 1 mmol L^−1^ MgCl_2_, and 1 mmol L^−1^ EDTA. Finally, the pellets were heated prior to immunoblotting analysis.

### Duolink In Situ PLA

Duolink in situ PLA was performed using a Duolink PLA kit (Sigma‒Aldrich) to further detect the association between IRF4 and KPNA6 in vivo. ≈5 × 10^5^ 3T3‐L1 cells were seeded in confocal dishes and cultured without antibiotics for 48 h. The cells were then fixed in 4% paraformaldehyde for 15 min at room temperature, washed with PBS twice for 20 min each, permeabilized with 0.2% Triton X‐100, blocked with Duolink blocking buffer for 30 min at 37 °C, and finally incubated with a mouse anti‐IRF4 mAb and a rabbit anti‐KPNA6 mAb. The PLA was subsequently performed in accordance with the manufacturer's instructions, and the signals were detected using a laser scanning confocal microscope (Zeiss LSM 800) with a 20× objective.

### GST Pulldown

For the GST pulldown assay, the full‐length sequence of IRF4 was cloned and inserted into pGEX‐6T‐1 (GE) containing a GST tag, and the full‐length sequence of KPNA6 was cloned and inserted into pET‐32a containing a His tag.

The plasmids for GST‐IRF4 and His‐KPNA6 were transfected into *Escherichia coli*. The fusion proteins were prepared as described previously. ≈100 µg of GST and GST‐IRF4 fusion protein was immobilized in 50 µL of glutathione agarose and equilibrated before being incubated together at 4 °C for 60 min with gentle rocking. ≈100 µg of His‐KPNA6 fusion protein was added to the immobilized GST‐IRF4 and GST after 3 washes with PBS with Tween 20 (PBST), and the two fusion proteins were incubated overnight at 4 °C with gentle rotation. The bound proteins were eluted with elution buffer (10 mM glutathione in PBS, pH 8.0) and analyzed by immunoblotting.

### Luciferase Assay

*Ucp1* (both the wild‐type and mutant) was constructed by digestion of the pmir‐PGLO vector (Addgene, Watertown, MA, USA) with two restriction enzymes (*Xhol I* and *Xbal I*), followed by the ligation of sequences encoding the corresponding 3′UTRs of the target genes. 3T3‐L1 cells were transfected with one of the abovementioned plasmids using Lipofectamine 2000 (Invitrogen) according to the manufacturer's instructions. After 48 h of transfection, the cells were lysed, and the luciferase activity was measured with a dual‐luciferase reporter assay kit (Vazyme, Nanjing, China). The data are presented as the ratio of Renilla luciferase activity to firefly luciferase activity.

### ChIP Assay

The ChIP experiments were performed using a ChIP Assay Kit (Millipore, #17‐10086) in strict accordance with the manufacturer's instructions. WAT obtained from *AAV‐oe‐βFaar* or control mice were fixed with 37% formaldehyde for 10 min and then subjected to chromatin fragmentation via 30 cycles of sonication lasting 3 s each. Chromatin was incubated overnight at 4 °C with an anti‐IRF4 antibody (#4964, CST) and subsequently immunoprecipitated using Proteinase K (Millipore). Purified DNA was amplified via PCR using primer pairs spanning the predicted *Irf4* binding sites on the *Ucp1* promoter. Table S2 (Supporting Information) provides a list of the sequences of the primers used in this process.

### Statistical Analysis

All in vivo experiments used individual mice as biological replicates, and the exact values of *n* are reported in the figure legends. The in vitro cell assays were performed in triplicate, and each experiment was independently repeated at least three times. The specific number of samples (*n*) is provided in the figure captions. The data are presented as the means ± standard errors of the means (SEMs). Statistical comparisons between two groups were performed using Student's *t*‐test, whereas ANOVA was employed for multiple group comparisons. For one‐way ANOVA, Dunn's multiple comparisons test was utilized, and Fisher's least significant difference (LSD) test was applied for two‐way ANOVA. Values of * *p* < 0.05, ** *p* < 0.01, and *** *p* < 0.001 were considered to indicate statistical significance. GraphPad Prism 8 software (GraphPad, San Diego, CA, USA) was used for all the statistical analyses.

## Conflict of Interest

There are no potential conflicts of interest relevant to this article to report.

## Author Contributions

Y.Y., B.H., and B.S. contributed equally to this work. Y.Y., B.H., B.S., and D.G. performed the experiments; Z.L., Y.Q., and Y.J. performed some of the animal experiments; X.C. collected the human samples; Y.P., Y.Z., and Y.S. analyzed the data; Y.L., F.Z., and L.J. designed the project; and F.Z. and L.J. interpreted the data and wrote the manuscript.

## Supporting information



Supporting Information

## Data Availability

Yes, we confirm.
