# Supplementary material for: The Long Noncoding RNA ΒFaar Promotes White Adipose Tissue Browning and Prevents Diet‐Induced Obesity
Source: Adv Sci (Weinh). 2025 Jun 23;12(35):e05545. doi: 10.1002/advs.202505545 (PMC12463088; doi:10.1002/advs.202505545)
Supplement: Supplementary file 1 — Supporting Information [file ADVS-12-e05545-s001.docx]

**the Long Noncoding RNA *ΒFaar* Promotes White Adipose Tissue Browning and**

**Prevents Diet-Induced Obesity**

Yue Yang^1#^, Bin Huang^1#^, Baixue Sha^#1^, Danni Gao^1^, Yimeng Qin^1^, Ziyi Li^1^, Xi Chen^1^, Yinuo Jin^4^, Yi Pan^1^, Yanfeng Zhang^1^, Yumeng Shen^2^, Yu Liu^3*^, Liang Jin^1*^, Fangfang Zhang^1*^

**Supplementary Figures:**

Figure S1

Figure S2

Figure S3

Figure S4

Figure S5

Figure S6

Figure S7

**Supplementary Spreadsheets:**

Table S1

Table S2

Table S3

Table S4

Table S5


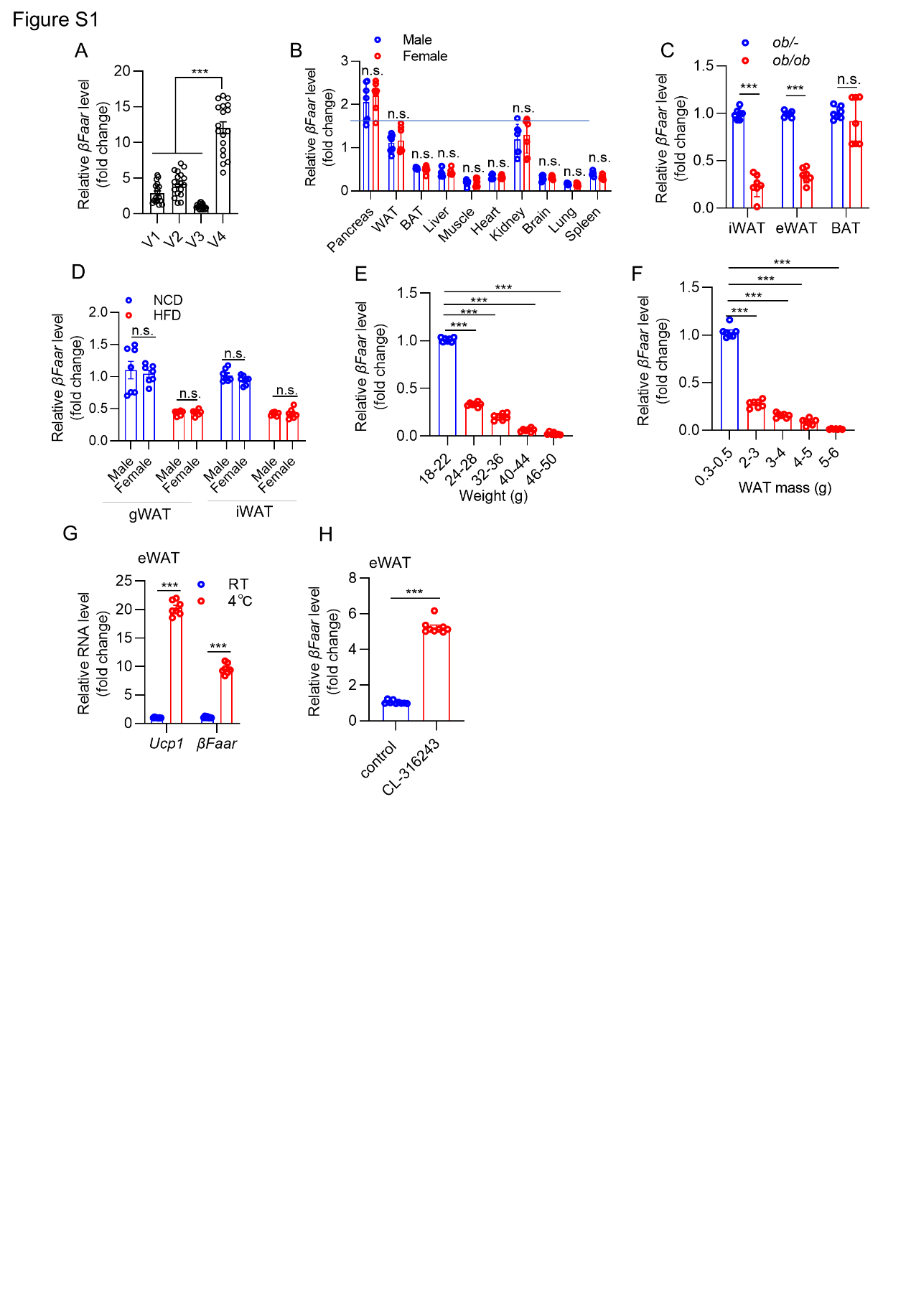


**Figure S1** (A) The expression levels of the four Has*-βFAAR* variants were quantified via qRT‒PCR (*n*=18 individuals). (B) Expression levels of *βFaar* were measured in different mouse tissues of female and male mice quantified via qRT‒PCR (*n*=7 mice). (C) The abundance of *βFaar* was assessed in iWAT, eWAT, and BAT of *ob/ob* mice or *ob/-* mice was quantified via qRT‒PCR (*n*=7 mice). (D) The expression levels of *βFaar* were tested in the gWAT and iWAT of female and male mice (*n*=7 mice). (E-F) The expression levels of *βFaar* were examined at different stages during obesity development in *ob/ob* mice (*n*=7 mice). (G) Treatment with low temperature for 24 h resulted in altered expression of *βFaar* and *Ucp1* in eWAT (*n*=7 mice). (H) Treatment with the β3 adrenoceptor agonist CL-316243 led to changes in the expression levels of *βFaar* in eWAT (*n*=9 mice). The fold change values were calculated via the 2^-ΔΔCt^ method. The data are presented as the means ± SEMs. The *p* values obtained using a two-tailed unpaired Student’s t test or two-way ANOVA are indicated; *** *p*<0.001.


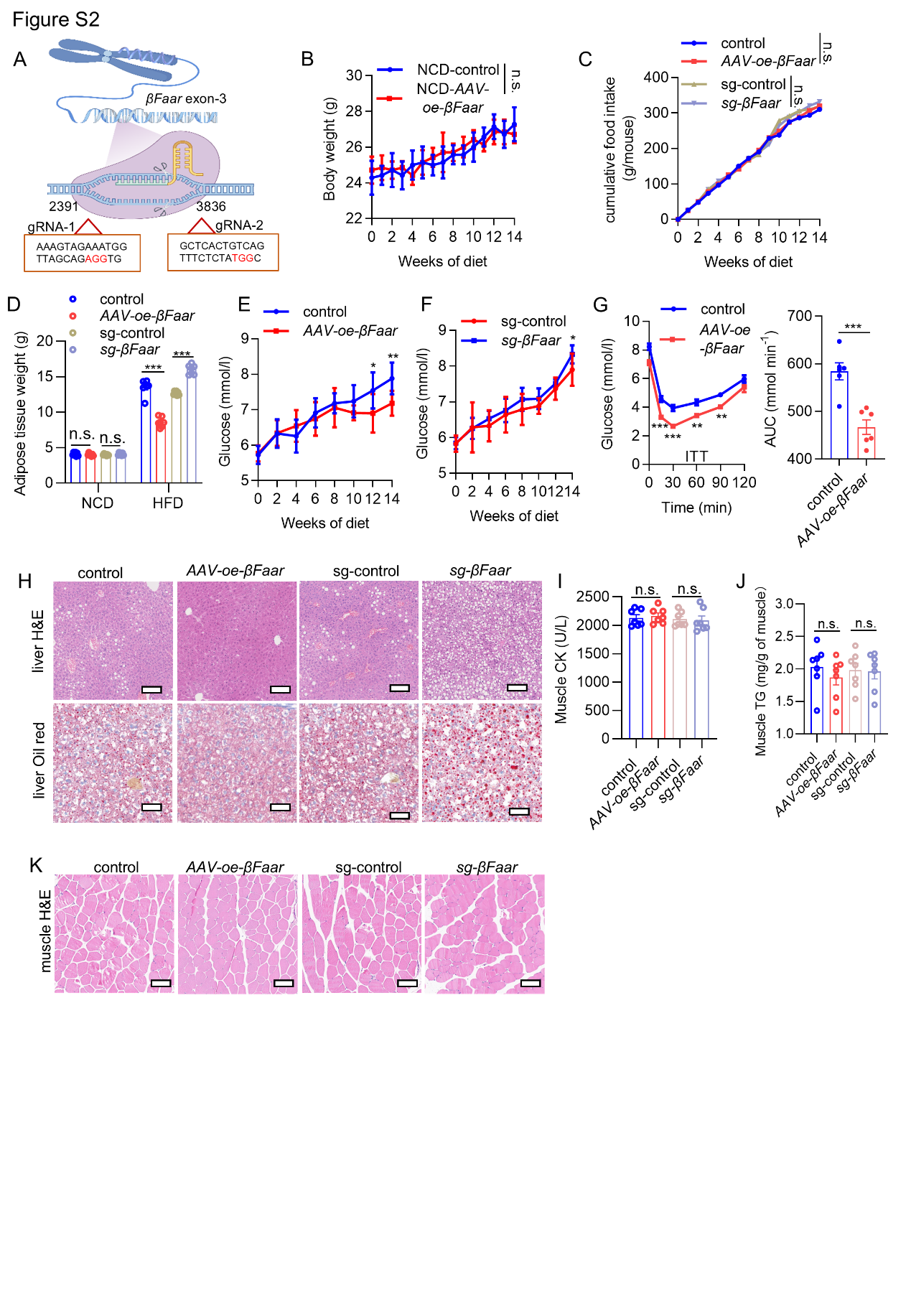


**Figure S2** (A) Generation of *βFaar*-KO mice by CRISPR/Cas9. Knockout strategy with gRNA sequences (gRNA-1 and gRNA-2), and relative positions of primers (F1/R1) used for genomic PCR and primers (F2/R2) used for detecting deletions. (B) Dynamic changes in body weight of AAV-*βFaar* mice and control mice with the 14 weeks of normal diet administered (*n*=7 mice). Schematic illustration was drawn by figdraw. (C) Monitored the regulatory effect of *βFaar* on food intake in HFD mice (*n*=7 mice). (D) The eWAT or iWAT weight of AAV-*βFaar* mice, *sg-βFaar* mice and control mice treated with NCD or HFD (*n*=7 mice)*.* (E-F) Dynamic changes in blood glucose concentration levels of *AAV-βFaar* (E, *n*=7 mice) *or sg-βFaar* (F, *n* =7 mice) treated with HFD. (G) IPITT (0.75 U/kg) was performed on both *AAV-βFaar* mice and control mice at week 14 of HFD administration, the corresponding area under the curve (AUC) of blood glucose level was calculated (*n*=6 mice). (H) Representative images of H&E staining and quantification of oil red O staining (bottom) in liver of *AAV*-*βFaar* mice and *sg*-*βFaar* mice (*n*=7 mice). Scale bar: 40 μm. (I-J) The content of CK (I) and triphosphate (J) were detected in muscle of *AAV*-*βFaar* mice and *sg*-*βFaar* mice (*n*=7 mice). (K) Representative images of H&E staining in muscle of *AAV*-*βFaar* mice and *sg*-*βFaar* mice (*n*=7 mice). Scale bar: 40 μm. The fold change in mRNA expression was calculated via the 2^-ΔΔCt^ method. The data are presented as the means ± SEMs. The *p* values obtained using a two-tailed unpaired Student’s *t* test or two-way ANOVA are indicated; ** p*<0.05, *** p*<0.01, **** p*<0.001.


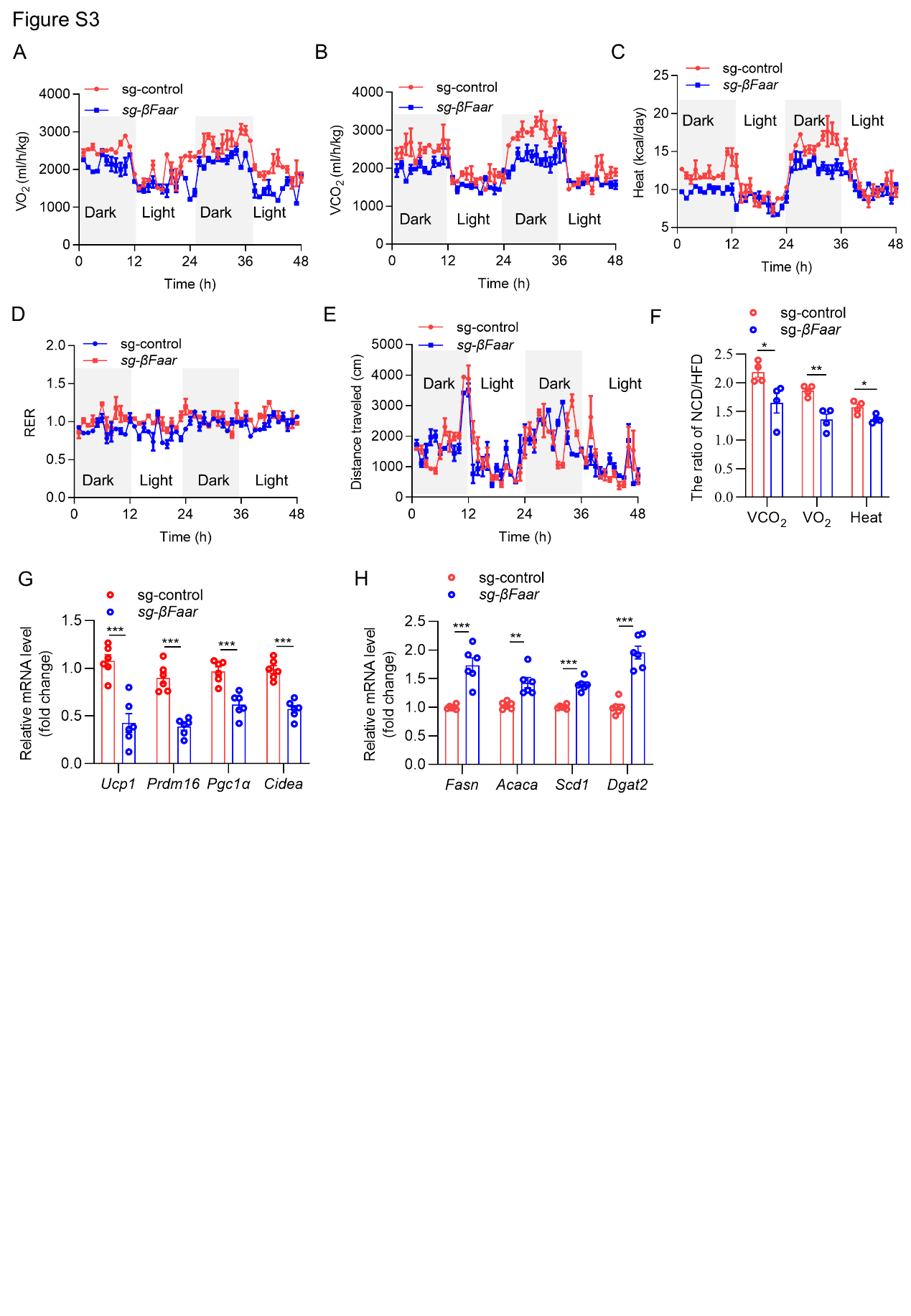


**Figure S3** (A-F) The variables of interest, including VO_2_ (A), VCO_2_ (B), heat production (C), the ratio of VCO_2_/VO_2_ (D), and average values of physical activity (E), were analyzed using *sg-βFaar*. The day/night bar represents a 12-hour duration. VO_2_, VCO_2_, and heat production were analyzed by ANCOVA with total body mass as a covariate (*n*=3 mice). (F) The ratio of O_2_ consumption, CO_2_ production, and heat generation were detected in HFD mice (*n*=3 mice). (G) Relative mRNA levels of thermogenic and lipolytic genes in *sg-βFaar* mice and the control group were quantified using qRT-PCR (*n*=3 mice). (H) Relative mRNA levels of adipogenic genes in *sg-βFaar* mice and the control group were quantified using qRT-PCR (*n*=3 mice). The fold change in mRNA expression was calculated via the 2^-ΔΔCt^ method. The data are presented as the means ± SEMs. The *p* values obtained using a two-tailed unpaired Student’s *t* test or two-way ANOVA are indicated; * *p*<0.05, ** *p*<0.01, *** *p*<0.001.


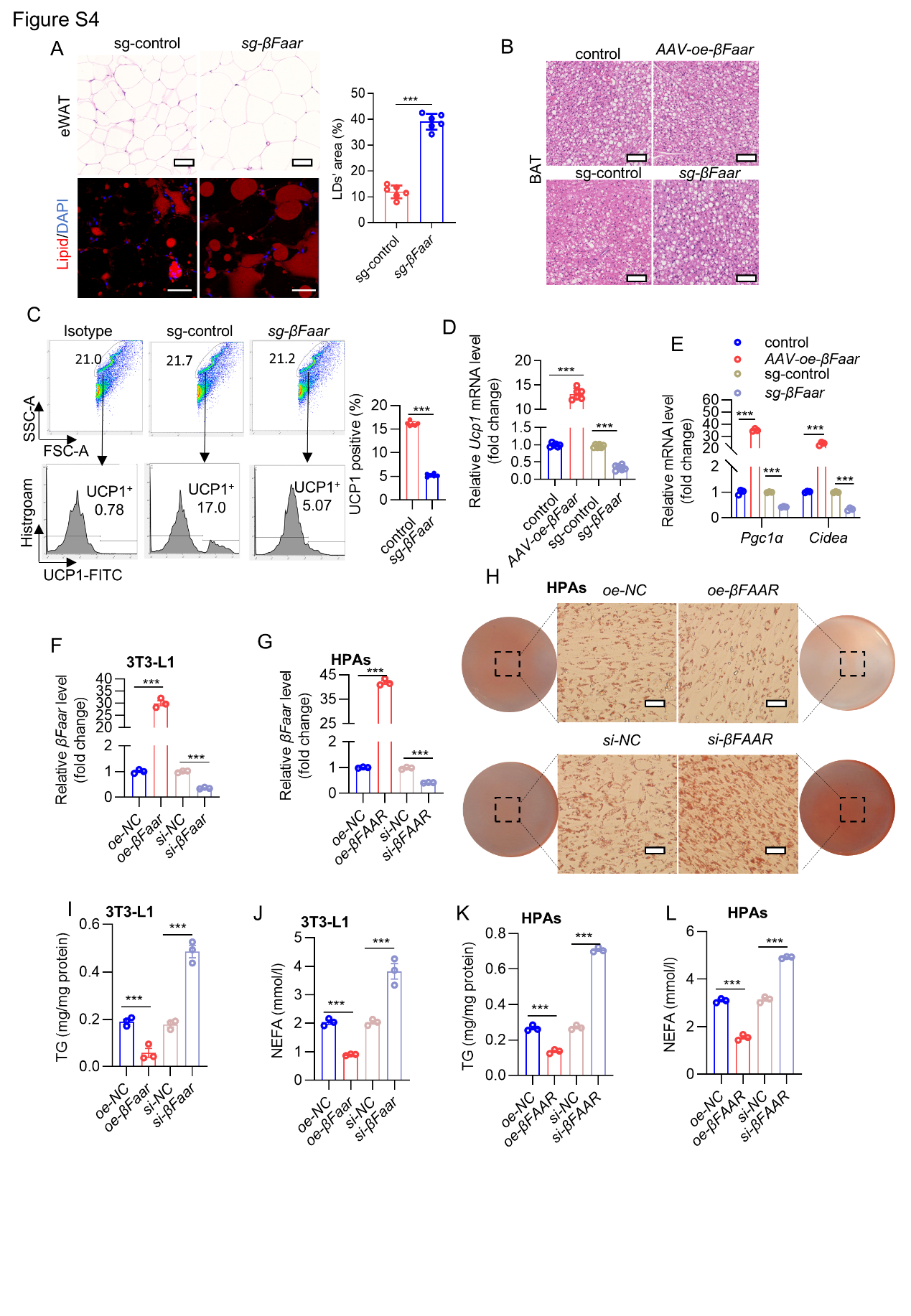


**Figure S4** (A) The images illustrating H&E staining in BAT sections with a scale bar of 100 μm (*n*=7 mice). (B) The images illustrating H&E staining and lipid immunofluorescence in eWAT sections treated with *sg-βFaar* or *sg-control* were obtained, the LDs’ area (%) was calculated in the right. H&E staining with a scale bar of 100 μm, lipid immunofluorescence with a scale bar of 20 μm (*n*=7 mcie). (C) Flow cytometry assay was employed to detect the content of UCP1^+^ cells in iWAT after treating with *sg-βFaar* or sg-control (*n*=7 mice). (D) qRT-PCR was performed to test the *Upc1* mRNA in the iWAT of *AAV-βFaar and sg-βFaar* mice. (E) Relative mRNA levels of *Pgc1α* and *Cidea* in iWAT of *AAV*-*βFaar or sg-βFaar* mice compared to the control group (*n*=3 mice). (F-G) The transfection efficiency of *βFaar* in the 3T3-L1 cells (F) and HPAs (G). (H) Oil red O staining was performed to evaluate the number of LDs present in differentiated human adipose primary cells transfected with *oe/si-βFAAR* or oe/si-control with a scale bar of 20 μm. (I, J) The content of TG (I) and NEFA (J) were quantified in the 3T3-L1 cells after transfected with *oe/si-βFaar* by using biochemical testing reagents. (K-L) The content of TG (K) and NEFA (L) were quantified in the HPAs cells after transfected with *oe/si-βFAAR* by using biochemical testing reagents. The fold change in mRNA expression was calculated via the 2^-ΔΔCt^ method. The data are presented as the means ± SEMs. The *p* values obtained using a two-tailed unpaired Student’s *t* test or two-way ANOVA are indicated; *** *p*<0.001.


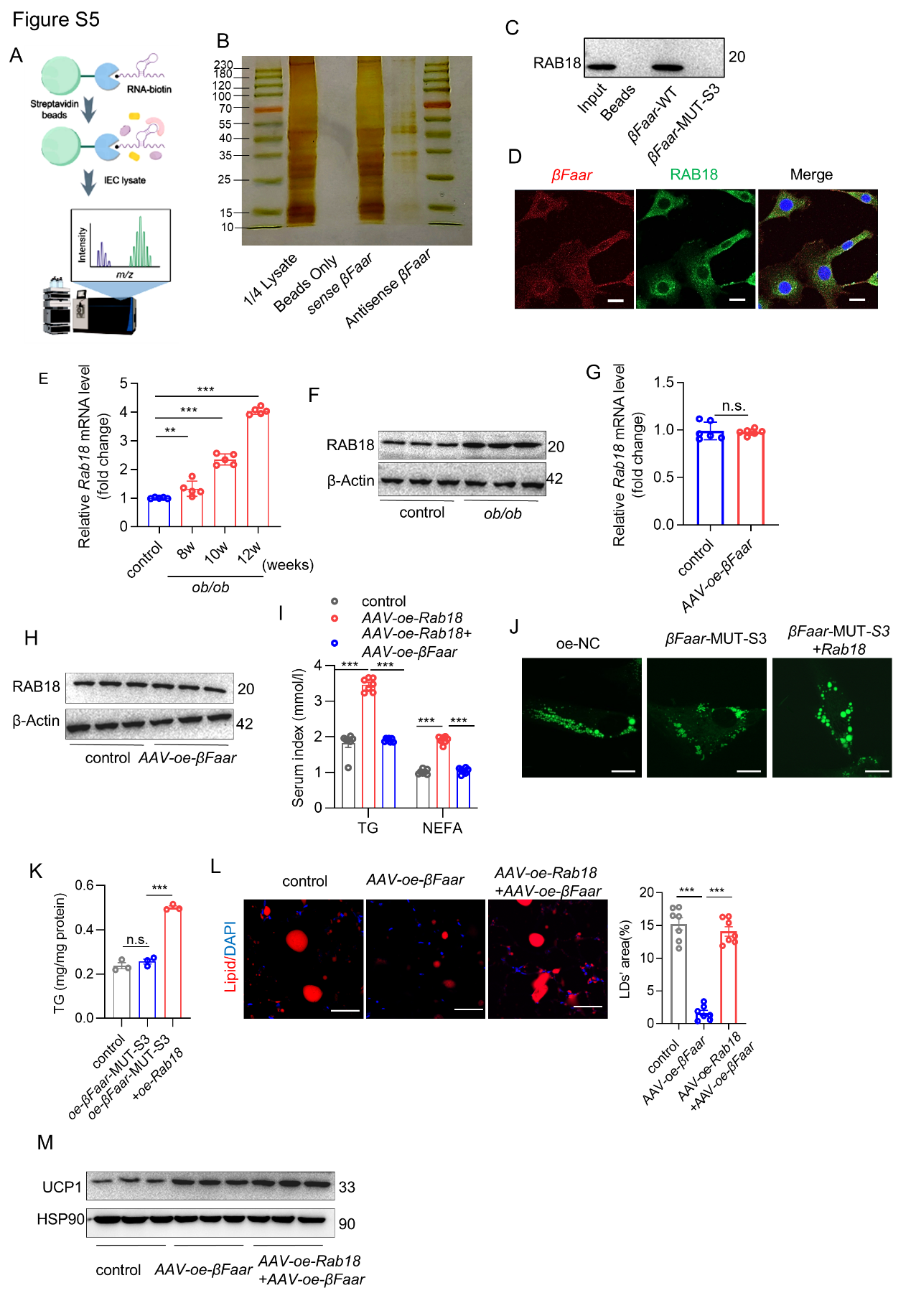


**Figure S5** (A) Schematic of RNA-protein pull down assays in 3T3-L1 cells lysates in combination with mass spectrometry analysis. (B) RNA pulldown assays were performed using biotin-labeled sense or antisense of *βFaar*. Silver staining was performed to identify the interacting proteins. (C) The 851-1248 nt of *βFaar* was mutant in accordance with the principle of indirect non-complementary pairing (*βFaar-*MUT-S3*)*. Then immunoblotting analysis revealed representative images displaying the interaction between RAB18 and *βFaar*-MUT-S2 in the pulldown assay. (D) Colocalization of *βFaar* (red) and RAB18 (green)was visualized by FISH/IF assays, with a scale bar indicating 40 μm. (E-F) Immunoblotting analysis and qRT-PCR revealed the protein and mRNA levels of RAB18 in the WAT of *ob/ob* mice (*n*=5-7 mice). (G-H) Immunoblotting analysis and qRT-PCR revealed the protein and mRNA levels of RAB18 in the eWAT after treated with *AAV-βFaar* (*n*=6-7 mice). (I) The content of serum levels of TG and NEFA, were detected in AAV-*Rab18*, AAV-*βFaar* and AAV-*Rab18* & AAV-*βFaar* mice (*n*=7 mice). (J) Representative images depicted LDs labeled green in *βFaar*-MUT-S3 *or βFaar-*MUT-S3*+Rab18*-transfected 3T3-L1 preadipocytes, with a scale bar indicating 20 μm. (K) the TG content in the 3T3-L1 cells transfected with *βFaar-*MUT-S3 *or βFaar*-MUT-S3*+Rab18*. (L) The lipid immunofluorescence treated with *AAV-βFaar*, *AAV-βFaar+AAV-Rab18* or control were obtained in iWAT, with a scale bar of 20 μm (*n*=7 mice). (M) Immunoblotting analysis revealed representative images displaying the expression levels of UCP1 treated with *AAV-βFaar* or *AAV-Rab18* & *AAV-βFaar* (*n*=6 mice). The fold change in mRNA expression was calculated via the 2^-ΔΔCt^ method. The data are presented as the means ± SEMs. The *p* values obtained using a two-tailed unpaired Student’s *t* test or two-way ANOVA are indicated; *** *p*<0.001.


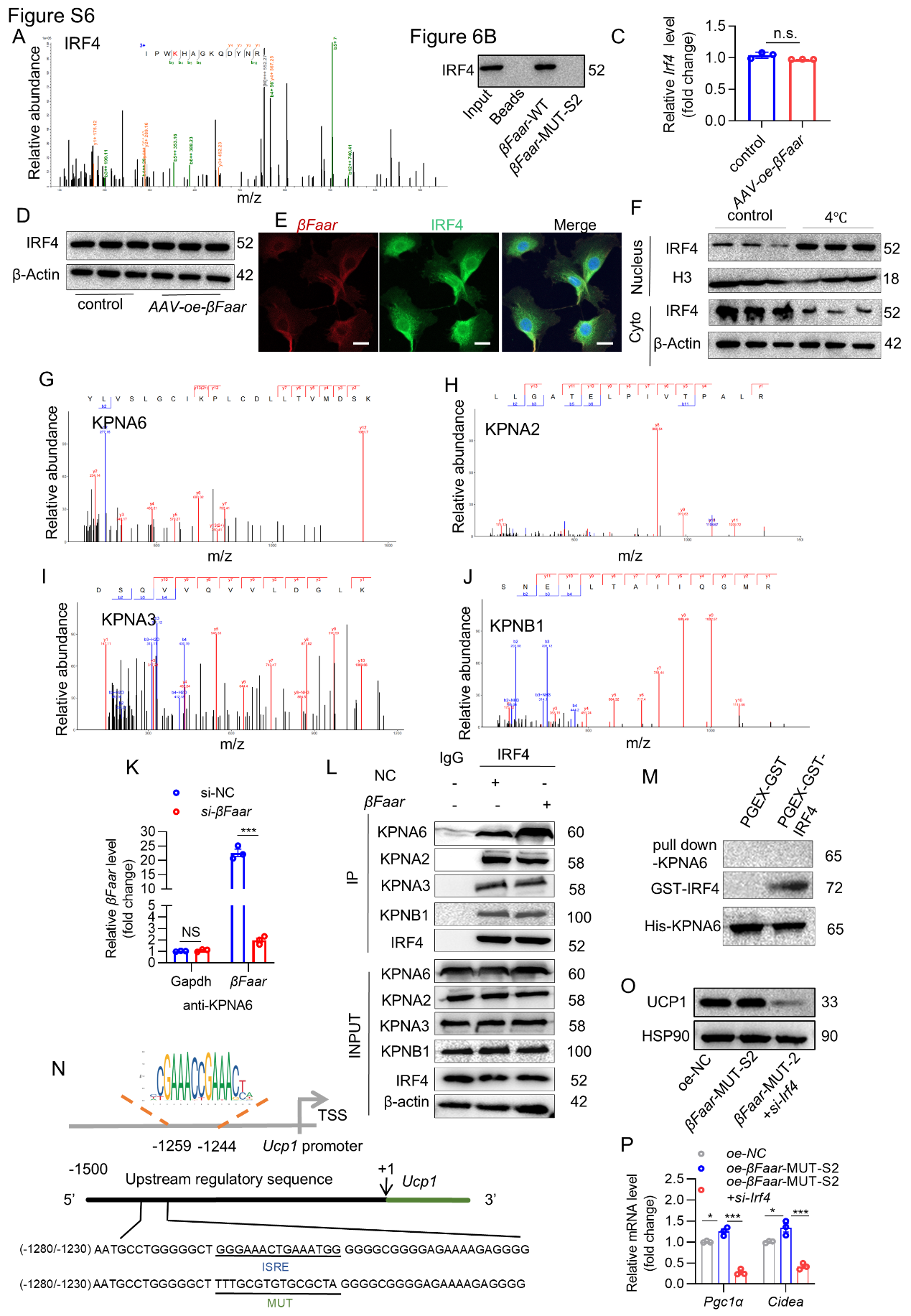


**Figure S6** (A) RNA pulldown assays were conducted using biotin-labeled sense or antisense probes targeting *βFaar*. Mass spectrometry (MS) profiles of the IRF4 band captured by *βFaar* were obtained, and the corresponding peptide sequences are presented above the graphs. (B) The 401-851 nt of *βFaar* was mutant in accordance with the principle of indirect non-complementary pairing (*βFaar-*MUT-S2*)*. Then immunoblotting analysis revealed representative images displaying the interaction between IRF4 and *βFaar*-MUT-S2 in the pulldown assay. (C, D) Immunoblotting analysis and qRT-PCR revealed the protein and mRNA levels of IRF4 after *AAV-βFaar* treatment (*n*=7 mice). (E) Colocalization of *βFaar* (red) and IRF4 (green) was visualized by FISH/IF assays, with a scale bar indicating 40 μm. (F) Immunoblotting images depicted the subcellular distribution of IRF4 with cold exposure or control. (G-J) MS profiles of KPNA2, KPNA3, KPNA6, and KPNB1 bands retrieved by *βFaar* were generated, with the corresponding peptide sequences listed above each graph. (K) Anti-KPNA6 RNA immunoprecipitation (RIP) was performed on 3T3-L1 cell lysate, followed by qRT-PCR analysis to examine the precipitated RNAs. *Gapdh* served as a control for validating KPNA6–*βFaar* interaction. (L) Immunoblotting analysis was conducted on control and *oe-βFaar* 3T3-L1 cells to assess the interaction between IRF4 and KPNA6, KPNA2, KPNA3 and KPNB1 proteins. (M) Detection of His-IRF4 bound to GST-KPNA6 or GST in a GST pull-down assay. (N) The transcription factor IRF4 binding to *Ucp1* promoter region was predicted using Jaspar and Promo databases. (O-P) The UCP1 protein level (O) and the mRNA levels of hallmark genes (P) in the 3T3-L1 cells transfected with *βFaar*-MUT-S2 or *βFaar*-MUT-S2+*si-Irf4*. The fold change in mRNA expression was calculated via the 2^-ΔΔCt^ method. The data are presented as the means ± SEMs. The *p* values obtained using a two-tailed unpaired Student’s *t* test or two-way ANOVA are indicated; *** *p*<0.001.


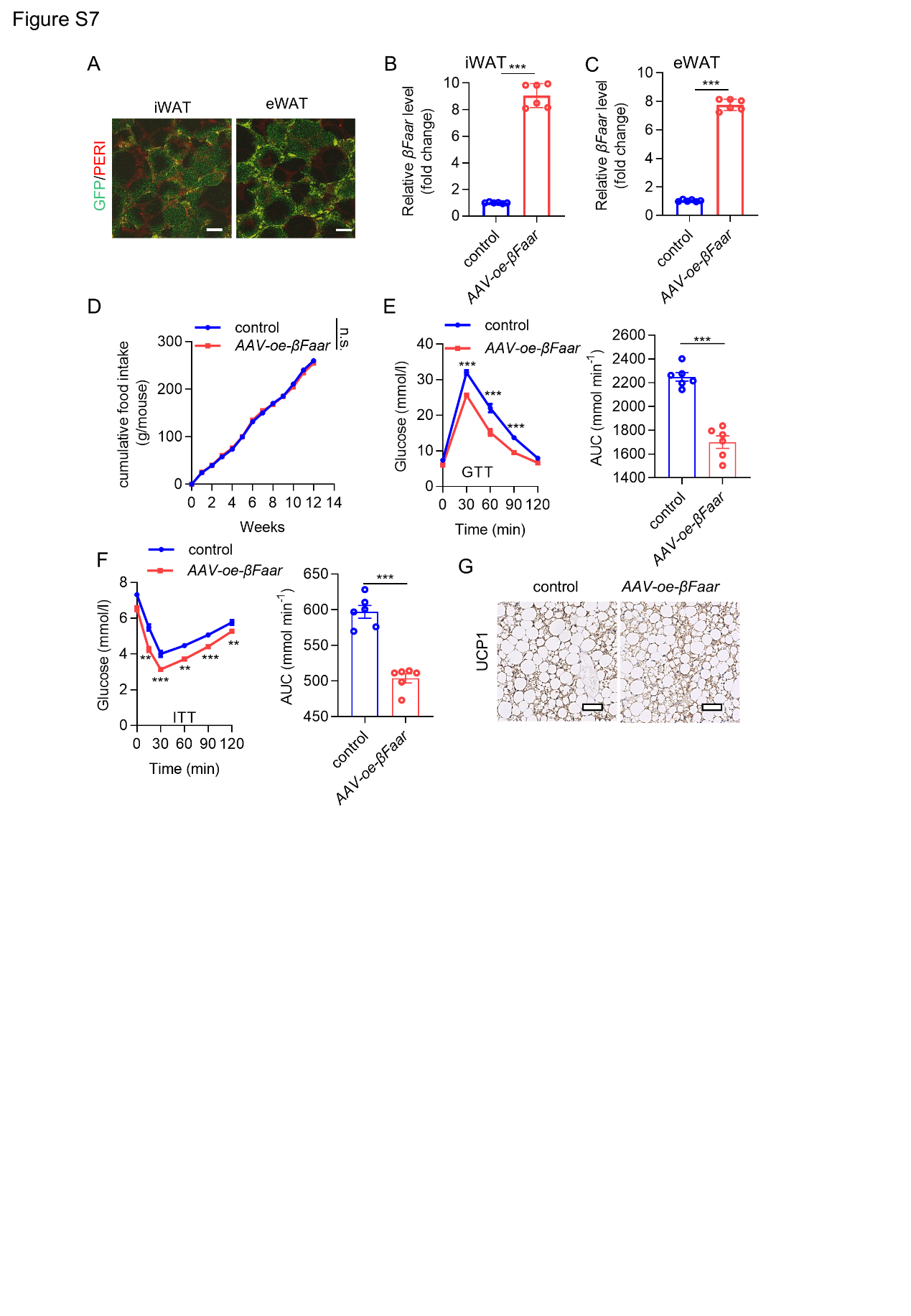


**Figure S7** (A) Histological analysis was performed to examine the GFP^+^ cells in iWAT and eWAT tissues transfected with control or *AAV-βFaar*. Perilipin (PERI) was utilized as a marker for identifying adipose tissues. Arrows indicate the presence of GFP^+^ cells, a scale bar of 40 μm is shown. (B, C) The expression level of *βFaa*r in iWAT and eWAT tissues was quantified using qRT-PCR (*n*=6 mice). (D) The regulatory effect of *βFaar* on food intake in HFD mice was monitored (*n*=10 mice). (E, F) IPGTT (E, 1.5 g/kg) and IPITT (F; 0.75 U/kg) were performed in *AAV-βFaar* mice and control mice at the 14th week of HFD administered, respectively. The corresponding area under the curve (AUC) of blood glucose level was calculated (*n*=6 mice). (G) Immunohistochemistry images demonstrated UCP1 staining in BAT sections, with a scale bar indicating 100 μm length scale reference. The fold change in mRNA expression was calculated via the 2^-ΔΔCt^ method. The data are presented as the means ± SEMs. The *p* values obtained using a two-tailed unpaired Student’s *t* test or two-way ANOVA are indicated; *** *p*<0.001.

Table S1 Clinical characteristics of the patients with obese patients and normal individuals

|  | Obesity | Normal | Total |
| --- | --- | --- | --- |
| Number（male/female） | 70 (45/55) | 30 (11/14) | 100 (56/69) |
| Age（years） | 34.60±11.90 | 41.50±12.40 | 36.50±11.80 |
| HOMA-IR | 3.95±1.34 | 0.19±0.04 | 2.91±1.55 |
| Glucose（mM） | 8.54±1.57 | 4.59±0.34 | 7.52±1.98 |
| BMI | 38.70±4.60 | 20.19±0.84 | 33.45±8.68 |

Table S2 The primers used in Real-time PCR (5’-3’)

| Gene | Forward Primer | Reverse Primer |
| --- | --- | --- |
| *βFaar* | TGCCGCGGAGAGGATATTTTT | TGGGGTCTGGTAGACATCCT |
| *Gapdh* | AGGTCGGTGTGAACGGATTTG | TGTAGACCATGTAGTTGAGGTCA |
| *U6* | CCACGAGGAAGAGAGGTAGC | CACTCAGACCGCGTTCTCTC |
| *Ucp1* | AGGCTTCCAGTACCATTAGGT | CTGAGTGAGGCAAAGCTGATTT |
| *Prdm16* | CCACCAGCGAGGACTTCAC | GGAGGACTCTCGTAGCTCGAA |
| *Fasn* | GGAGGTGGTGATAGCCGGTAT | TGGGTAATCCATAGAGCCCAG |
| *Acaca* | ATGGGCGGAATGGTCTCTTTC | TGGGGACCTTGTCTTCATCAT |
| *Acly* | CAGCCAAGGCAATTTCAGAGC | CTCGACGTTTGATTAACTGGTCT |
| *Acox1* | TAACTTCCTCACTCGAAGCCA | AGTTCCATGACCCATCTCTGTC |
| *Pparα* | AGAGCCCCATCTGTCCTCTC | ACTGGTAGTCTGCAAAACCAAA |
| *Pgc1α* | TATGGAGTGACATAGAGTGTGCT | CCACTTCAATCCACCCAGAAAG |
| *Cidea* | TGACATTCATGGGATTGCAGAC | GGCCAGTTGTGATGACTAAGAC |
| *Scd1* | TTCTTGCGATACACTCTGGTGC | CGGGATTGAATGTTCTTGTCGT |
| *Dgat2* | GCGCTACTTCCGAGACTACTT | GGGCCTTATGCCAGGAAACT |
| *Rab18* | TTTGCACGCAAGCATTCTATGT | TTGTTCTGGTTCTCACTTTCCC |
| *Irf4* | TCCGACAGTGGTTGATCGAC | CCTCACGATTGTAGTCCTGCTT |
| *Has-βFAAR V1* | AGTGGCGTTTGTCTTCATTCA | TGCCCAATACCACATGGAC |
| *Has-βFAAR V2* | AGATTTCCTTAAGCACATACTCC | TCATGTCTGTTCCCAGCAA |
| *Has-βFAAR V3* | TGAAGACTAATAGGCTCTGT | GGGCATCTATTGTTCACCA |
| *Has-βFAAR V4* | GAATCAGAAAGCCAACACC | CTACTCGTTTGGAACCGTTG |
| *Ucp1*-promoter CHIP | TAAGCAGCCTAGTGGTGG | CCACCACTAGGCTGCTTA |

Table S3 Primer sequences used for PCR

| Gene | Forward strand (5’-3’) | Reverse strand (5’-3’) |
| --- | --- | --- |
| *Rab18* | ATGGACGAGGACGTGCTG | TTATAGCACAGAGCAGTAACCG |
| *Irf4* | ATGAACTTGGAGACGGGCAGCC | TCACTCTTGGATGGAAGAATGACGG |

Table S4 Oligo sequences used for shRNA

| Gene | Forward strand (5’-3’) | Reverse strand (5’-3’) |
| --- | --- | --- |
| *sh-Rab18-1* | CATGCTAGTTGGAAATAAA | TTTATTTCCAACTAGCATG |
| *sh-Rab18-2* | CGATTTCAGTGGATGGAAA | TTTCCATCCACTGAAATCG |
| *sh-Rab18-3* | CGGTTACTGCTCTGTGCTA | TAGCACAGAGCAGTAACCG |
| *sh-Irf4-1* | GGTTGATCGACCAGATCGA | TCGATCTGGTCGATCAACC |
| *sh-Irf4-2* | CCTCCTACTTGGAAGACAA | TTGTCTTCCAAGTAGGAGG |
| *sh-Irf4-3* | GTATTACTTTGCTCAACAA | TTGTTGAGCAAAGTAATAC |

Table S5 Oligo sequences used for siRNA

| Gene | Sequence (5’-3’) |
| --- | --- |
| *βFaar*-siRNA-1 | GGTTTGAAGTGTTGTGTAT |
| *βFaar*-siRNA-2 | GACTGTCAGTCCTGGAGAA |
| *βFaar*-siRNA-3 | GTATGAGCAGAGAGCTATA |
| *βFaar*-ASO-1 | AAAAGCTGAGAAGCCAAGAC |
| *βFaar*-ASO-2 | GAACAGCCCACCTTTAAACA |
| *βFaar*-ASO-3 | GTATGAGCAGAGAGCTATA |
| *βFaar*-sgRNA-1 | AAAGTAGAAATGGTTAGCAG |
| *βFaar*-sgRNA-2 | GCTCACTGTCAGTTTCTCTA |
| *Has-βFAAR*-sgRNA-1 | GCGGCGGAGCACCGATCTCA |
| *Has-βFAAR*-sgRNA-2 | CTACTCGTTTGGAACCGTTG |
